# Interacting Qubit-Photon Bound States with Superconducting Circuits

**DOI:** 10.1103/physrevx.9.011021

**Published:** 2019

**Authors:** Neereja M. Sundaresan, Rex Lundgren, Guanyu Zhu, Alexey V. Gorshkov, Andrew A. Houck

**Affiliations:** Department of Electrical Engineering, Princeton University, Princeton, New Jersey 08544, USA; Joint Quantum Institute, NIST/University of Maryland, College Park, Maryland 20742, USA; Joint Quantum Institute and Joint Center for Quantum Information and Computer Science, NIST/University of Maryland, College Park, Maryland 20742, USA; Department of Electrical Engineering, Princeton University, Princeton, New Jersey 08544, USA

**Keywords:** Quantum Physics, Quantum Information

## Abstract

Qubits strongly coupled to a photonic crystal give rise to qubit-photon dressed bound states. These bound states, comprising the qubits and spatially localized photonic modes induced around the qubits, are the basis for many exotic physical scenarios. The localization of these states changes with qubit detuning from the photonic crystal band edge, offering an avenue of *in situ* control of bound-state interaction. Here, we present experimental results from a device with two transmon qubits coupled to a superconducting microwave photonic crystal and realize tunable on-site and interbound state interactions. We observe a fourth-order two-photon virtual process between bound states indicating strong coupling between the photonic crystal and transmon qubits. Because of their localization-dependent interaction, these states offer the ability to realize one-dimensional chains of bound states with tunable and potentially long-range interactions that preserve the qubits’ spatial organization. The widely tunable, strong, and robust interactions demonstrated with this system are promising benchmarks towards realizing larger, more complex systems that use bound states to build and study quantum spin models.

In the strong-coupling domain, a qubit coupled to a photonic band edge forms an exponentially localized photonic mode at the qubit position, which together with the qubit forms a qubit-photon dressed bound state [1–7]. Photonic crystals are natural avenues to realize these bound states due to their intrinsically tailorable band structure, and characteristic Bloch mode electric field distribution [8] which enables access to strong coupling with qubits [9–13]. Bound states in multiqubit photonic crystal devices are an ideal platform to study many-body quantum optics in one-dimensional systems [6,7,14–19]. Unlike many qubits coupled to a common cavity mode but similar to the case of some optical multimode cavities [20,21], coupling to a band edge creates bound states that intrinsically preserve the spatial organization of qubits, offering the ability to create one-dimensional chains of bound states with tunable and potentially long-range interactions. The promise of engineering interaction profiles beyond the intrinsic flip-flop with additional microwave drive tones further opens the possibility of simulating a wide range of quantum spin models in future devices [17]. In this paper, we demonstrate and characterize the underlying, fundamental tunable on-site and interbound-state interactions in a superconducting microwave photonic crystal device coupled to two transmon qubits.

A single dressed bound state, seeded by a single qubit in a crystal, is itself a unique avenue of study. Liu *et al*. first directly detected such a bound state in a stepped-impedance microwave crystal coupled to a single transmon qubit [13]. That work characterized the dependence of localization length on detuning between the transmon qubit and the band edge and further confirmed the existence of the localized state in the band gap when the bare transmon qubit is in the passband—an unmistakable signature of non-Markovianity, as seen in Figs. 1(e), 1(f), and Appendix D. We note that the transmission dip observed in Figs. 1(e) and 1(f) is due to the reflection from the transmon qubit [22–24]. State localization is tunable *in situ* with frequency through a range determined by device parameters, including transmon qubit-waveguide coupling and band curvature. Compared with previous work, we attain increased localization in this device [Fig. 1(b)] due mainly to a flatter band dispersion, realized by tailoring the unit cell of the photonic crystal (see Appendix A for a detailed discussion of the experimental parameters of our system). The bound-state localization length in this device is still widely tunable, which is critical for realizing strong, tunable interaction between spatially separated bound states. As the different coupling regimes translate to dramatically altered system behavior [7], it is important to determine which domain our system falls under. In systems such as the one presented here, qubit emission into the waveguide being larger than the other decay rates (coherent atom-photon interaction rates larger than decay rates) is the minimal coupling criterion, upon which the dressed bound state within the gap can be spectrally resolved [7]. The strong coupling criterion corresponds to the situation where a bare qubit resonant with the band edge gives rise to a bound state that is shifted from the band edge by more than the bound state’s linewidth [7,13]. In our finite system, we observe an approximately 250-MHz separation between the bound state and the band edge with bound-state linewidth of 4 MHz when a qubit is resonant with the band edge, thus firmly reaching the strong coupling condition [see Figs. 1(b), 1(e), and 1(f)]. By fabricating two transmon qubits in the photonic crystal [see Fig. 1(a) and Appendix B for a discussion on coupling transmons to photonic crystals], we realize multiple, spectrally resolvable bound states and can study interbound-state interaction.

The nature of interbound-state interaction makes this platform intrinsically well suited for investigating one-dimensional chains of bound states [see Fig. 1(c)]. Realizing sizable chains is possible by increasing the number of unit cells—a property that does not impact the Bloch mode distribution or band dispersion. Thus, qubits can be in separate unit cells but realize nearly identical coupling to the band edge. As the strength of interbound-state interaction depends on the spatial overlap of the photonic wave functions with the qubits, the distance separating qubits (set by device design) is directly mapped into the interactions of the system, maintaining the chainlike interaction pattern. Furthermore, in the investigation of bound states, the finite size of the crystal is a practical advantage: the overlaps of bound states with ends of the crystal lead to only quasi-bound states, allowing for detection through transmission measurements (see Appendix C 2 for a proof). Direct detection of such a state in this way was first demonstrated in Liu *et al*. [13].

Controlling photon-mediated interaction between superconducting qubits has been demonstrated in other one-dimensional systems—such as two qubits in a cavity [25,26] or in a linear waveguide [27]. However, in these cases the distance between the qubits was effectively eliminated (i.e., standing-wave interaction in a cavity) or otherwise reduced (modulo wavelength in a linear dispersion waveguide). Thus, photonic crystals and tunable bound states offer a fundamentally distinct form of interaction.

In addition to determining localization length, the frequency of the bound state also determines on-site interaction strength. In Figs. 2(a) and 2(b), we characterize the dependence of the transition frequencies between the three lowest levels of the bound state on bare transmon qubit frequency, and observe the steady reduction in bound-state anharmonicity from over 350 MHz to 0 MHz as the transmon qubit is tuned from deep in the band gap to the passband. Here, we have defined the bound-state anharmonicity as $\tilde{\Delta }=2{\tilde{\omega }}_{01}-{\tilde{\omega }}_{12}$, where ${\tilde{\omega }}_{01}$ and ${\tilde{\omega }}_{12}$ are the dressed bound-state frequencies [see level diagram in Fig. 2(a)]. This is dramatically more than the approximately 10% modification of transmon qubit anharmonicity with frequency expected when a transmon qubit is strongly coupled to a cavity mode [28].

Therefore, while we may treat the one-excitation and two-excitation bound states as first $\left(|\tilde{1}\rangle \right)$ and second $\left(|\tilde{2}\rangle \right)$ excited states of a new effective anharmonic transmon qubit [13], it is important to note that this effective transmon qubit differs in frequency and anharmonicity from the bare multilevel transmon qubit. Defining the three lowest bare transmon qubit levels as |0〉 |1〉, and |2〉, here the two-excitation bound is largely due to the coupling of the second transmon qubit transition (|1〉 ↔ |2〉 with the band edge rather other the multiphoton effects [7,15] (see Appendix F 1).

Numerical simulations, modeling the photonic crystal as a coupled cavity array with free parameters fit to match the band curvature from the dispersion relation [7,29] (see Appendix C for details), show similar dependence of anharmonicity on detuning [see Fig. 2(a) inset and Fig. 2(b)]. Unlike the transfer matrix method [30–32], this approach can extend beyond the single-excitation manifold to capture the higher levels of the bound state, as well as the Lamb shift of the qubit frequency, observed when including next-nearest-neighbor hopping between coupled cavities. Each transmon qubit is modeled as a three-level ladder (unless otherwise mentioned) with negative anharmonicity, and with the |0〉 ↔ |1〉 and |1〉 ↔ |2〉 transitions coupled with amplitudes *g* and $g\sqrt{2}$, respectively, to its local cavity site. It is critical to include level |2〉 to accurately reproduce the two-excitation manifold observed in experiment.

The tunable level structure also emerges in the emission spectrum of a continuously driven bound state [Figs. 2(c) and 2(d)], induced by a single qubit with bare frequency above the band edge. At low drive amplitude or Rabi frequency, transmission across the crystal via the bound state exhibits antibunching [see Fig. 2(c), inset] [33], consistent with single-photon transport of a two-level system and resonant pump (see Appendix D 4) [34–36]. As Rabi frequency is increased, we see a Mollow triplet emission spectrum [Fig. 2(c)], characteristic of a driven two-level system. When the Rabi frequency is on the same order as the anharmonicity, the bound state can no longer be approximated as a two-level system. In this domain, the steady state will be a mixture of the three eigenstates obtained by diagonalizing the drive Hamiltonian in the Hilbert space spanned by |0〉 |1〉, and |2〉. Transitions between all eigenstates result in six sidebands [37]. These six sidebands are visible in Fig. 2(d), though emission intensity varies greatly among them due to eigenstate population. A seventh transition is evident in the data [7.25 GHz in Fig. 2(d)]. This additional transition is due to the fourth effective transmon qubit level $\left(|\tilde{3}\rangle \right)$ while its curvature is reproduced by including a fifth effective transmon qubit level $\left(|\tilde{4}\rangle \right)$ in our numerical simulations (see Appendix E). Crucial to reproducing this transition in our theoretical simulations is taking into account that the bound-state level structure cannot be defined by a single anharmonicity, i.e., given the anharmonicity $\tilde{\Delta }={\tilde{\omega }}_{12}-{\tilde{\omega }}_{01}$ of the bound state, the frequency of the fourth level of the bound state is not simply given by $3{\tilde{\omega }}_{01}-3\tilde{\Delta }$ as is expected for a transmon [38] [see transmon level diagram in Fig. 2(a)].

We observe the flip-flop interaction (by a flip-flop interaction between two qubits with basis states |0〉, |1〉, we mean a Hamiltonian proportional to |01〉〈10| + |10〉〈01|) between the two the spatially bound states measuring the avoided crossing in transmission when the bound states are tuned into resonance. As these qubits (while we really have a multilevel systems coupled to the photonic crystal, we refer to them as qubits when one can ignore higher levels) are a fixed distance apart (9 mm) and there is negligible direct capacitive coupling, the strength of the flip-flop interaction will be entirely determined by the overlap of the localized photonic mode of one qubit with the other qubit, controllable here via the qubit frequencies.

In Fig. 3(a), the frequency of one qubit is held constant while the other is tuned through resonance. Measuring transmission at the single-photon level reveals an avoided crossing between the $|\tilde{0}\tilde{1}\rangle$ and $|\tilde{1}\tilde{0}\rangle$ levels of the coupled dressed bound states. Transmission amplitude of a bound state dims when the bound state and bare qubit are near resonance [see Fig. 3(a) inset]. From this plot, we can extract a resonant bound-state–bound-state interaction of 120 MHz for a 7.73-GHz bare qubit frequency. In comparison, Figs. 3(b)–3(d) show reduced interaction strength when both qubits are further detuned from the band edge, 6.125 GHz, 6.75 GHz, and 7.625 GHz, respectively, for the fixed qubit frequency.

To characterize this aspect of the two bound-state interaction, we map the magnitude of the avoided crossing as a function of detuning. In Fig. 3(g), the qubits are maintained on resonance with one another while being simultaneously tuned through the band gap. Theoretical modeling [Figs. 3(g) and 8(e)] shows experimental data to be consistent with localized photonic states and with interaction via wave-function overlap. In the limit where the qubit is deep in the gap, the Markovian approximation holds as is evident in the probability that the bare qubits are in the excited state (obtained from the hopping model) in Fig. 3(g) [see Fig. 8(c) for the photonic probability of the bound states]. Here, the localized mode and flip-flop interaction both have the same distance dependence *e*^−*x/L*^ where $L=a\sqrt{\alpha /\delta }$, is the localization *δ* is the detuning between the bare qubit and the band edge, *a* is the unit cell size, and the band-edge dispersion is *ω*_*k*_ = *ω*_0_ + *αa*^2^(*k* − k_0_)^2^ (see Appendix D 2). The corresponding flip-flop interaction Hamiltonian is $H\propto \sum_{j,l}S_{i}^{+}S_{j}^{-}{\left(-1\right)}^{\left|x_{i}-x_{j}\right|/a}e^{-\left|x_{i}-x_{j}\right|/L}$. When the bare qubit frequencies are near the band edge the probability that the bare qubits are in the excited state decreases [Fig. 3(g)] and we have non-Markovian behavior (see Appendix D 3 for a detailed on the breakdown of Markovian behavior). While our experiment studies steady states, in other settings, where an initial state evolves in the absence of an input field, non-Markovianity can lead to the preservation of entanglement during time evolution [6,39–41]. It would be interesting to study in future work whether there is also entanglement in steady state and how to best measure it in our system.

We now turn to the qubit nature of the bound states when the bare qubit frequencies are resonant. The qubit part of the wave function of these two bound states (obtained from the effective Hamiltonian of the system) is (approximately) either symmetric (|0〉|1〉 + |1〉|0〉) or antisymmetric (|0〉|1〉 − |1〉|0〉) We theoretically predict that the higher (lower) frequency bound state at resonance in Fig. 3(a) is symmetric (antisymmetric) (see Appendix D 2). This turns out to affect transmission through the system. Intuitively, the antisymmetric state is expected to be dimmed as the exponential parts of the localized photonic states cancel each other out; and hence the linewidth, which is proportional to the wave function at the end of the photonic crystal, is smaller. However, because the band edge is not at zero momentum in our system, it turns out the symmetric state is actually dimmed and has a smaller linewidth, as we prove in Appendix D 2. In Fig. 3(e), we see that the bound states at the same transmission frequency (with different bare qubit frequencies) have drastically different linewidths with the higher-frequency bound state having a smaller linewidth, consistent with our numerical simulations [Figs. 3(f) and 8(d)]. This provides some indirect experimental evidence that the qubit part of the higher (lower) frequency bound-state wave function is indeed symmetric (antisymmetric).

To further study tunable on-site interaction, we probe the interacting bound states beyond the one-excitation manifold using spectroscopic measurements [see Fig. 4(a)]. Similar to spectroscopy of qubits in cavities, we can use transmission at the band edge to help detect bound-state transitions, a technique that provides sharper contrast compared to transmission measurement for the more highly localized bound states and allows detection of higher-dressed transitions, such as the transition between |0〉 and |2〉 driven by two photons of frequency *ω*_02_=2.

With this technique we detect interaction between |02〉, |20〉, and |11〉 of the coupled bound states, observed as avoided level crossings. In addition to the single-photon exchange interaction between |02〉 |20〉 |11〉 and [26], remarkably we measure the two-photon virtual interaction between |20〉 and |02〉, despite the fact that this process is fourth order in coupling *g* (see Appendix F 2). This two-photon interaction shows consistent dependence on detuning: increasing in strength (from 0 MHz to over 10 MHz) as the bound states shift towards the band edge and the states become more delocalized [see inset of Fig. 4(a)]. Numerical simulations [Fig. 4(b)] are consistent with experimental data and capture the relative magnitudes of interaction between levels as well as frequency dependence on coupling strengths. Observation of this small interaction highlights the overall strength of interbound-state coupling possible via overlap alone.

The widely tunable on-site and interbound-state interactions demonstrated with this device and consistent theoretical simulations are promising benchmarks towards realizing larger, more complex systems of bound states. Examples of these systems include spin models with both local and long-range interactions, which arise when the bare qubit frequencies are deep in the band gap [17], and complicated multiqubit-photon bound states, which arise when the bare qubit frequencies are in the passband (see, e.g., Ref. [7]). Beyond stepped impedance coplanar waveguides, there are undoubtedly numerous ways to realize superconducting microwave photonic crystals, including lumped element or Josephson junction-based designs, that are equally compatible with superconducting qubits. Regardless of the platform, behavior of bound states due to qubit-band edge coupling will mirror the behavior characterized in this work—elevating this platform above any single experimental design choice.

While the bound states were centered in neighboring unit cells in this device, this is not a limitation or requirement for future experiments as the range of localization can be accordingly set via the basic crystal parameters, as seen by comparing bound-state linewidths measured here with those reported previously [13]. Therefore, one can realize a one-dimensional chain of bound states in a moderately sized photonic crystal, where individual control over the qubits would allow dialing up or down long-distance interaction between sets of qubits.

In this work the interactions were *in situ* tunable via qubit frequency (DC flux), a static quantity on the timescale of the bound-state lifetime. Dynamically controllable interactions would introduce an additional tool for designing and manipulating spin Hamiltonians [17]. One method for realizing this type of fast timescale control is flux pumping, a technique involving microwave frequency modulation of the qubit frequency along the flux bias line [42–45]. Another potential pathway would use an auxiliary microwave field through the crystal itself. Here, the qubits could be maintained on resonance deep in the band gap such that there is minimal interaction via bound-state overlap. A single rf control tone can be turned on to induce a transition close to the passband, thus redressing the bound states into new, effective bound states with interaction strength depending on properties of the microwave drive. The addition of several drives or precisely shaped microwave pulses (made possible by commercial high-speed arbitrary microwave waveform generators [46]) promise not only changing interaction strength but also modifying the shape of the interaction itself—from an exponential to a sum of exponentials—leading to a wide range of possibilities including power-law-decaying interactions [17]. These supplementary forms of tunable control would expand the ability of the qubit-photonic crystal platform to realize a broader class of tunable spin models.

## Figures and Tables

**FIG. 1.**
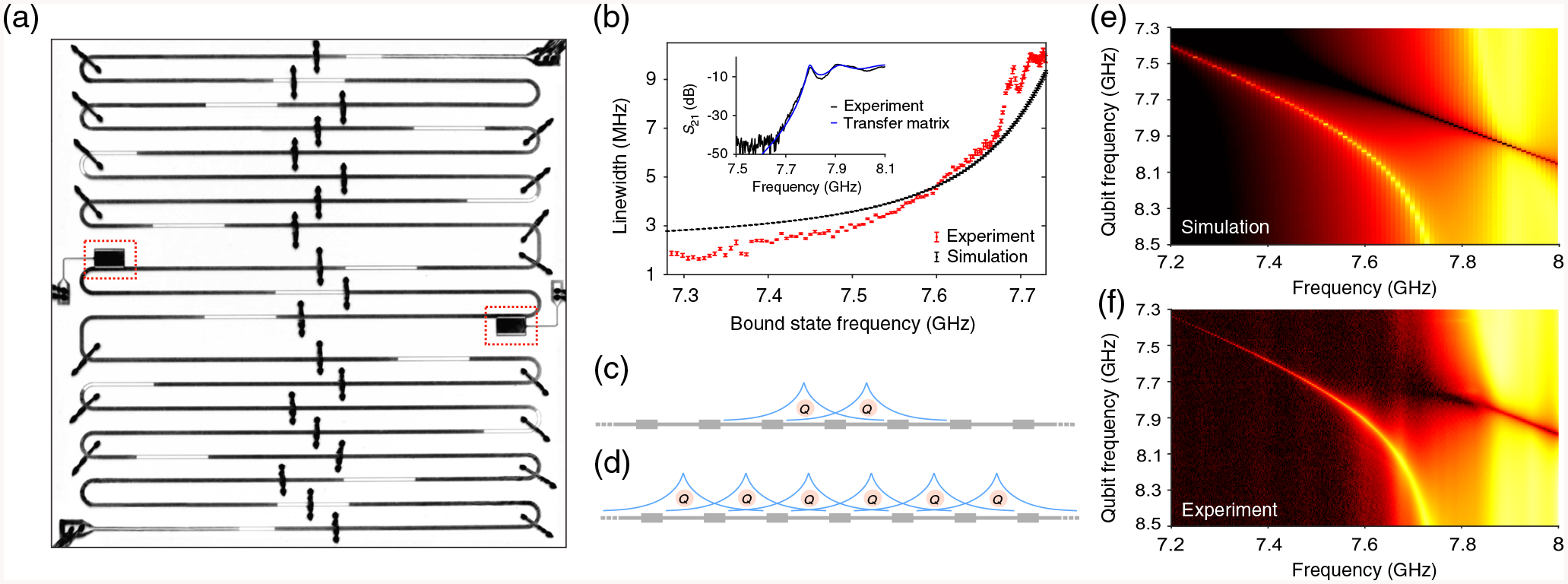
A platform for interacting dressed bound states.— (a) A 16-site microwave photonic crystal is realized by alternating sections of high and low impedance coplanar waveguide. Two transmon qubits (multilevel, anharmonic energy ladder) are in neighboring unit cells in the middle of the device, centered in the high impedance sections for maximal coupling to the band edge at 7.8 GHz [all values presented in units of (2*π*) Hz, i.e., *ω*_BE_ = 7.8 (2*π*) GHz]. For this experiment, the passband (band gap) refers to states above (below) the band-edge frequency. Each transmon is individually tunable in frequency via a local flux bias line. (b) Bound-state linewidth, an indirect measure of localization, varies with bare transmon qubit frequency. The wide range over which photon localization can be tuned indicates the feasibility of realizing a chain of strongly interacting bound states. Experimentally measured and simulated linewidths are shown in red and black, respectively. Inset: Overlay of simulated *S*_21_ from the transfer matrix method (blue) and measured high-power *S*_21_ (black) shows good agreement in bare crystal characteristics. (c) The interaction between bound states will be determined by overlap of their localized photonic envelopes with the qubits. (d) One can couple more qubits to the band edge by adding them to other cells of the photonic crystal. In such a system, the localization-length-dependent interaction of the bound states would preserve the spatial organization of qubits across the crystal, and determine the many-body structure of the interactions. (e) Experimental data and (f) hopping model simulation for *S*_21_ vs single-qubit frequency and probe frequency. The bare band edge is at 7.797 GHz. The bright peak in the band gap is the dressed qubit-photon bound state. The bound state always exists within the band gap for qubit frequencies (the other qubit is far detuned and has negligible effect) both above and below the band edge—a clear signature of non-Markovianity.

**FIG. 2.**
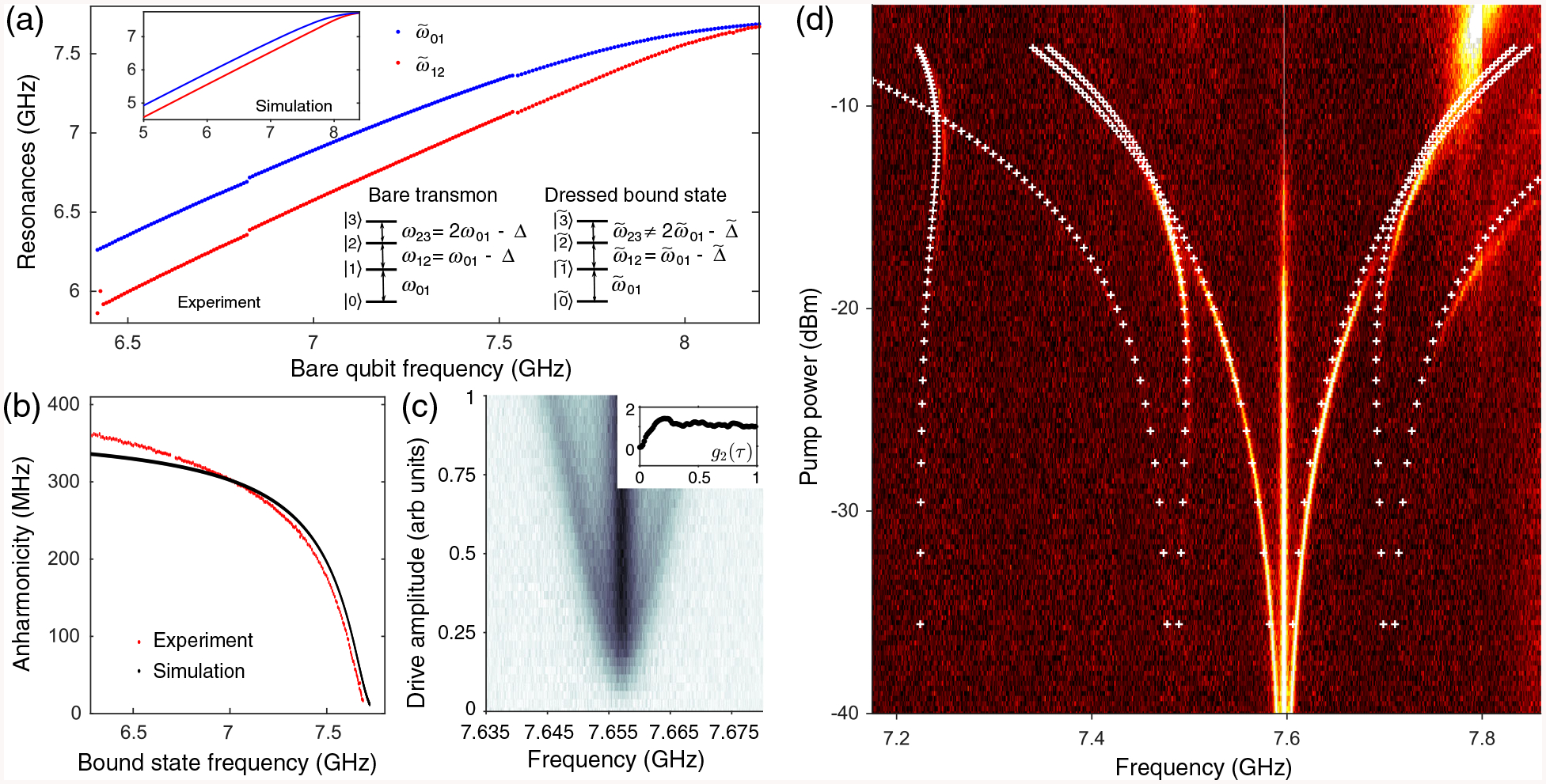
Probing the bound-state energy levels.— (a) The anharmonicity of the bound state $\tilde{\Delta }$ is dependent on bare transmon qubit frequency *ω*_01_, demonstrating a tunable on-site interaction strength. In blue (red), the first (second) transition of the bound state is measured across a range of bare qubit frequencies (inset: simulation). Upper left corner: level diagram of the bare transmon and dressed transmon. (b) Decreasing anharmonicity with increasing bound-state frequency shown in red for experimental data and black for simulation. (c) Power spectrum of a resonantly driven bound state for increasing drive amplitude. Sidebands are linearly displaced from the central peak with increasing drive amplitude, characteristic of the Mollow triplet. Inset: second-order autocorrelation measurement for drive amplitude = 0.2 is consistent with single photon, antibunched transport. (d) Emission spectrum of a resonantly driven (≈7.59 GHz) bound state (induced by a qubit at 7.9 GHz, which is above the band edge located at 7.8 GHz) as a function of drive power. At low drive power, only the Mollow triplet is observed. With increasing power we see four additional sidebands, two on either side of the original Rabi sidebands, which together are the transitions between the three lowest levels of the anharmonic bound state (|0〉, |1〉, |2〉). The white crosses are from numerical simulations (see Appendix E). We have included five transmon qubit levels in our simulation. See text for the discussion of the seventh sideband around 7.25 GHz.

**FIG. 3.**
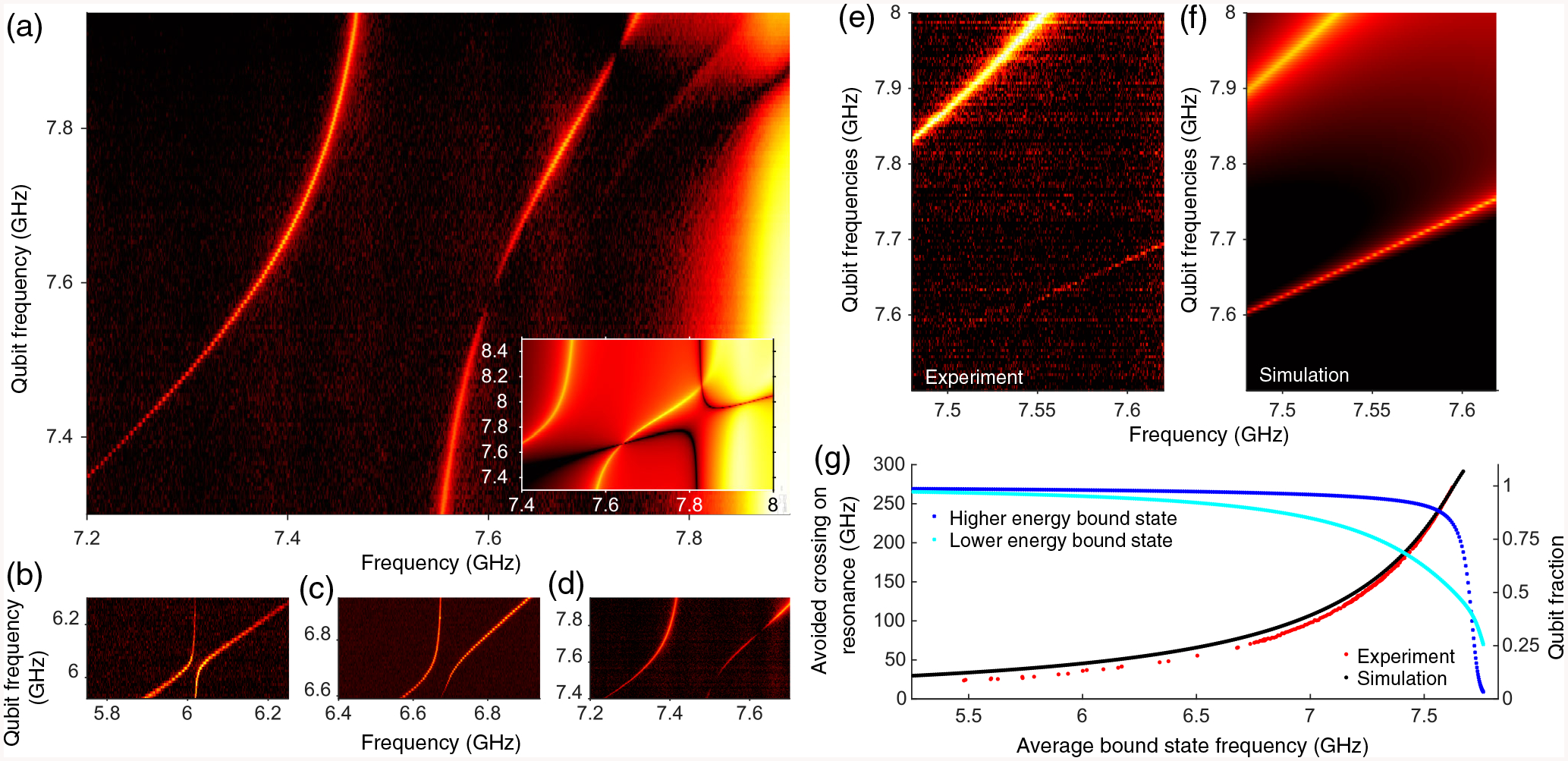
Interacting bound states.— Interaction between bound states is characterized by the avoided crossing (observed in *S*_21_ measurement) that arises while tuning one qubit (*y* axis) through resonance with the other (fixed). (a) An avoided crossing of 240 MHz is observed when the fixed qubit is at 7.73 GHz. The two points where transmission amplitude of a bound state dims are understood as the bound-state peak being resonant with the qubit frequency. (a), inset—Hopping model simulation of the one-excitation manifold is consistent with experimental observation. The lamb shift in the hopping model originates from next-nearest-neighbor interaction between coupled cavities. (b),(c),(d) Tunable bound-state interaction strength is illustrated in example bound-state avoided level crossings for a fixed qubit whose bare frequency is circa 6.125, 6.75, and 7.625 GHz. As qubits are detuned further from the band edge, bound states are more tightly localized, reducing overlap and thus reducing interaction. (e),(f) Transmission when the qubits are on resonance across a range of qubit frequencies in the experiment and the simulation, respectively. The uneven linewidths of the two bound states when they occur at the same frequency suggest they are symmetric (higher-frequency bound state) and antisymmetric (lower-frequency bound state) states (see main text). (g) Bound-state avoided crossing and qubit population (from simulation) as a function of average bound-state frequency. A steady reduction in interaction strength occurs with increasing detuning from the band edge (moving deeper into the band gap) due to increasing localization of the bound states. Hopping model simulation (black) captures this detuning-dependent behavior observed in experiment (red). Near the band edge, both bound states (blue and cyan) have a significant photonic contribution.

**FIG. 4.**
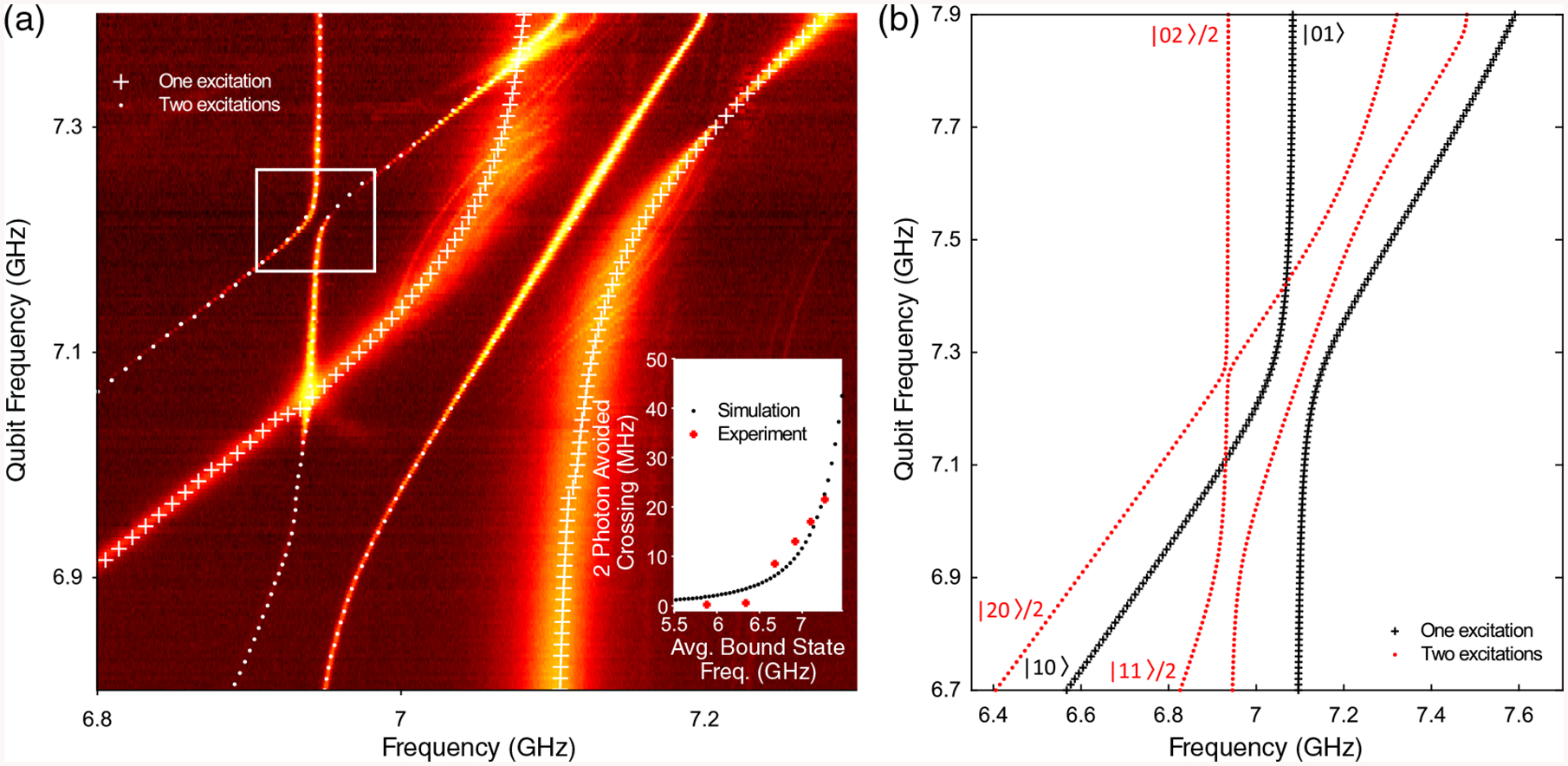
Interaction between two-excitation levels of two bound states.— (a) Spectroscopic measurement while tuning one bound state through the other (qubit fixed at 7.2 GHz), reveals survival of strong interaction into the two-excitation manifold. Crossed and dotted lines are guides to the eye to discern the levels belonging to the first (|01〉 and |10〉) and second (|02〉, |20〉, and |11〉) excitation manifolds, respectively. Here, we have labeled our states as |transmon one; transmon two〉. In addition to the |02〉(|20〉) and |11〉 avoided level crossing, we also detect a two-photon virtual interaction between |02〉 and |20〉 white box). This interaction—fourth order in coupling *g*—manifests itself in avoided level crossings up to and exceeding 20 MHz. For comparison, the |02〉(|20〉)-|11〉 and |01〉-|10〉 interactions are second order in *g* and thus both are significantly stronger. Inset: two-photon avoided crossing strength versus average bound-state frequency. (b) Numerical simulation for fixed bare qubit frequency of 7.27 GHz, with the one (two) excitation manifold in black (red).

**FIG. 5.**
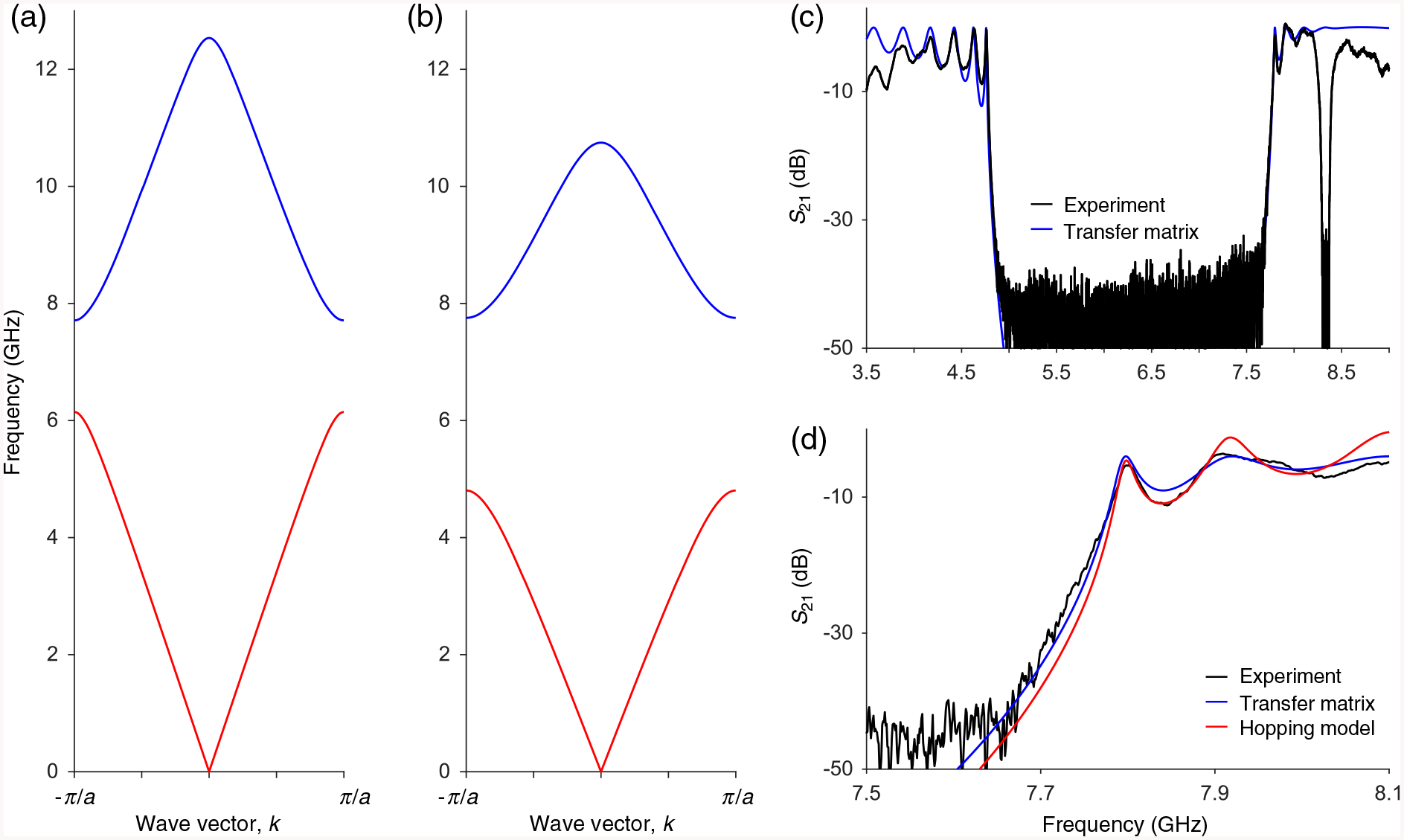
First two bands of simulated dispersion for *Z*_lo_ = 25 Ω, *Z*_hi_ = 124 Ω and (a) *L*_lo_ 0.45 mm, *L*_hi_ 8 mm (parameters from [13]), and (b) *L*_lo_ = 1.2 mm, *L*_hi_ = 7.8 mm (parameters from present paper). (c) Overlay of simulated *S*_21_ from the transfer matrix method [blue; same parameters as (b)] and measured high-power *S*_21_ (black) shows good agreement in bare crystal characteristics. (d) Overlay of simulated *S*_21_ from the transfer matrix method [blue; same parameters as (b)] and from the hopping model [red; with *κ* = 1 GHz and *κ*_0_ = 4 MHz] and measured high-power *S*_21_ (black) near the band edge. Drop in transmission circa 8.3 GHz is due to TWPA dispersion, not device defect.

**FIG. 6.**
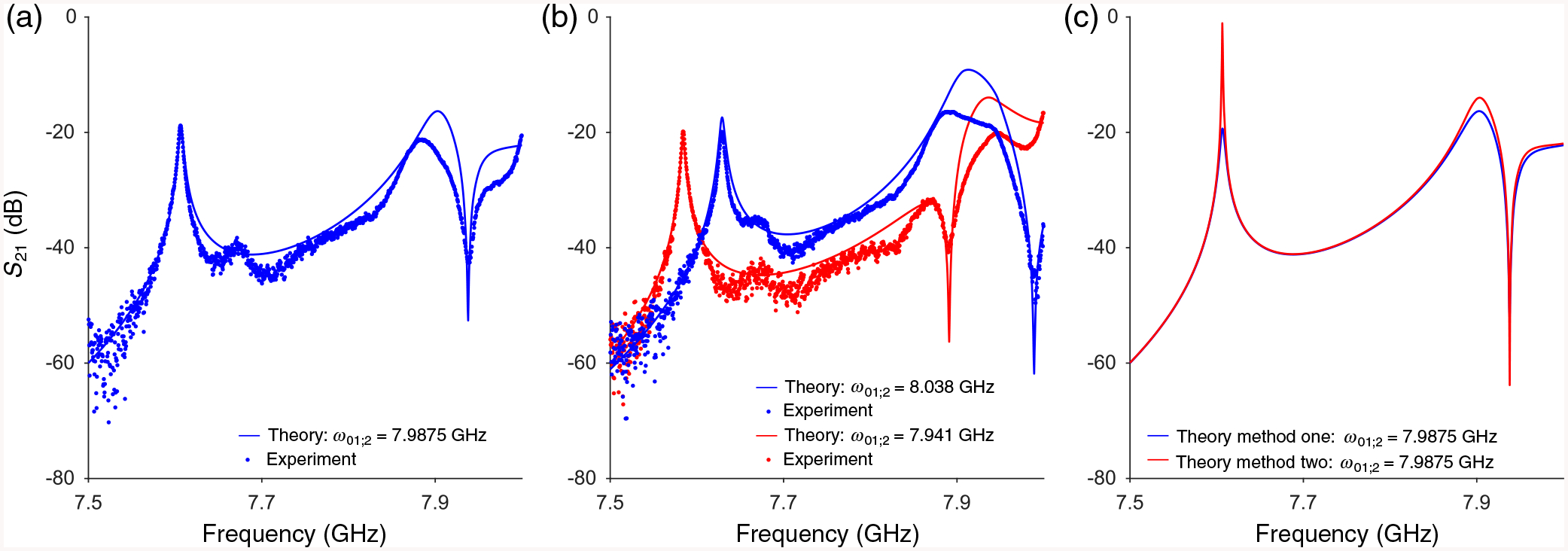
Dependence of transmission *S*_21_ on drive frequency, dependence used for determining the coupling strength of the second qubit, i.e., the one at site *N*/2 + 1 = 9. (a) Solid blue line is theoretical data for *ω*_01;2_ = 7.9875 GHz while blue dots are experimental data. We choose parameters of the Hamiltonian such that the bound-state frequency, the transmission dip frequency, and the linewidth of the bound state match the experimental data. (b) Solid blue line is theoretical data for *ω*_01;2_ = 7.941 GHz and blue dots are experimental data. Solid red line is theoretical data for *ω*_01;2_ = 8.038 GHz and red dots are experimental data. (c) Comparison of transmission methods for ω_01;2_ = 7.9875 GHz. The solid blue line is from method one, while the solid red line is from method two. The bound-state and transmission dip occur at the same frequencies for both methods.

**FIG. 7.**
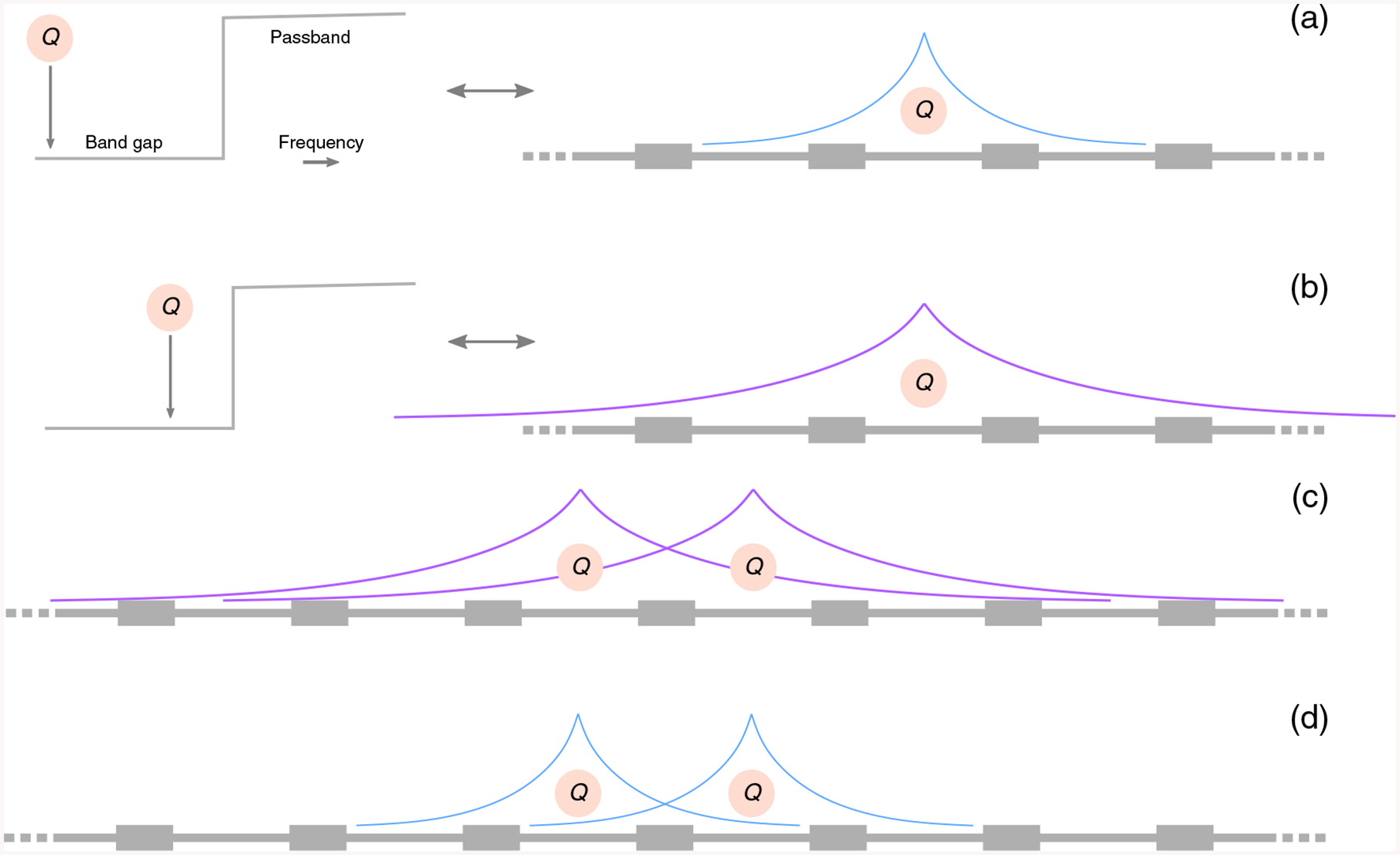
Visualizing bound states.— A qubit (pink circle) is coupled to one site of a 1D photonic crystal (gray line of alternating width). Coupling a qubit and band edge produces a photonic envelope (blue and purple) that is spatially centered at the qubit location. (a),(b) The localization of the photonic component is determined by the detuning of the qubit from the band edge. (a) is more localized than (b) as the detuning is larger in the former. The overlap of the photonic wave function with the ends of the crystal (not shown) determines the linewidth of the bound state measured in transmission. The strength of the interaction between the bound states, when qubits are resonant with one another, can be understood in (c) and (d) as depending on the localization of the bound states.

**FIG. 8.**
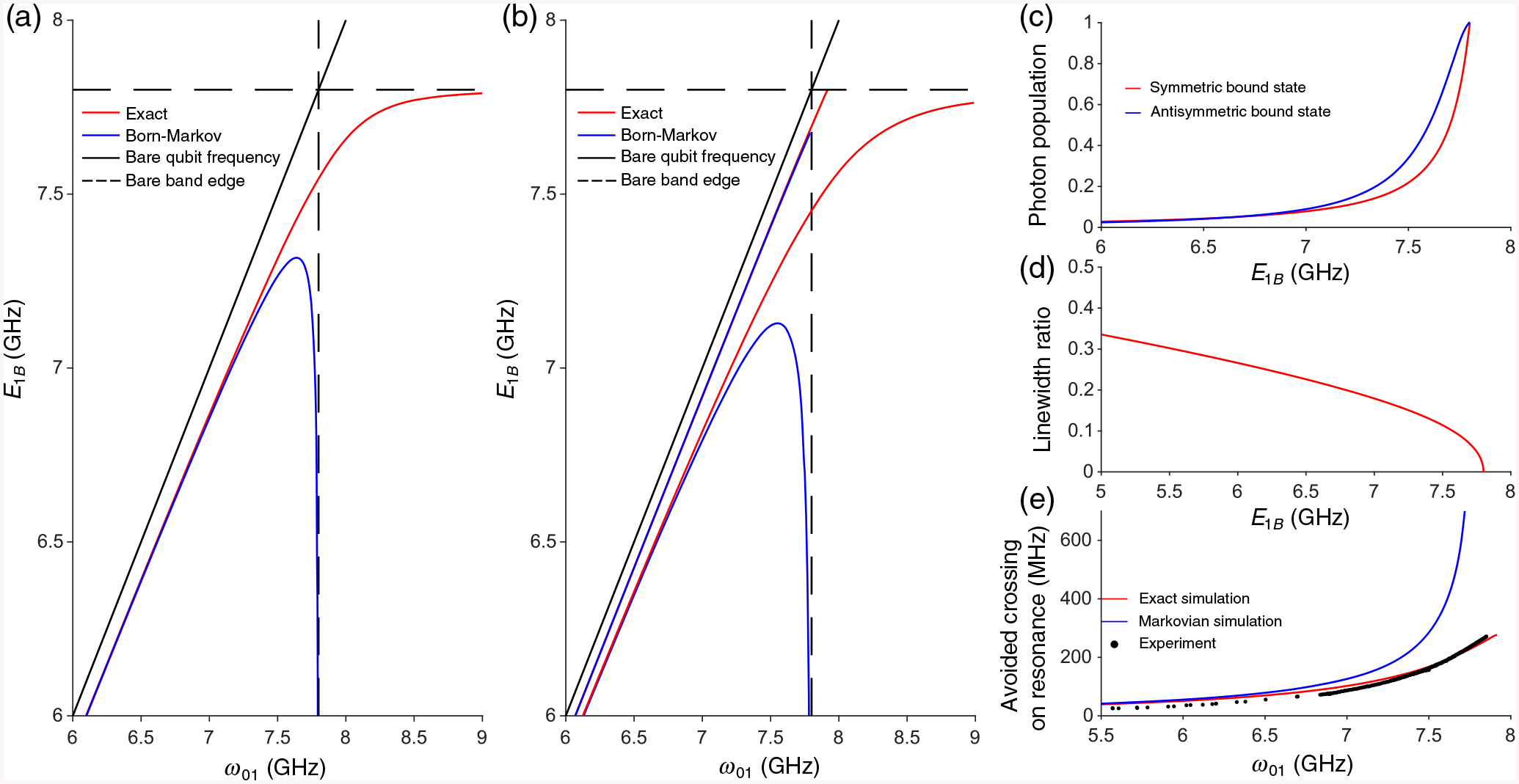
Exact solution versus Born-Markov approximation. Here, the red lines are the exact solution while the blue lines are the Born-Markov approximation. The dashed black lines mark the band edge and the thick black line is the bare qubit frequency. Here, we take *ω*_0_ = 7.8 GHz, *α* = 1.155 GHz, and *g* = 0.5275 GHz. (a) Single qubit. (b) Two qubits for |z_1_ − z_2_| = 1. (c) Theoretical photon population of the bound states. (d) Linewidth ratio of the two bound states as a function of bound-state energy. (e) Avoided crossing on resonance versus bare qubit frequency. We see that the experimental data are well described by the Markovian solution only deep in the gap.

**FIG. 9.**
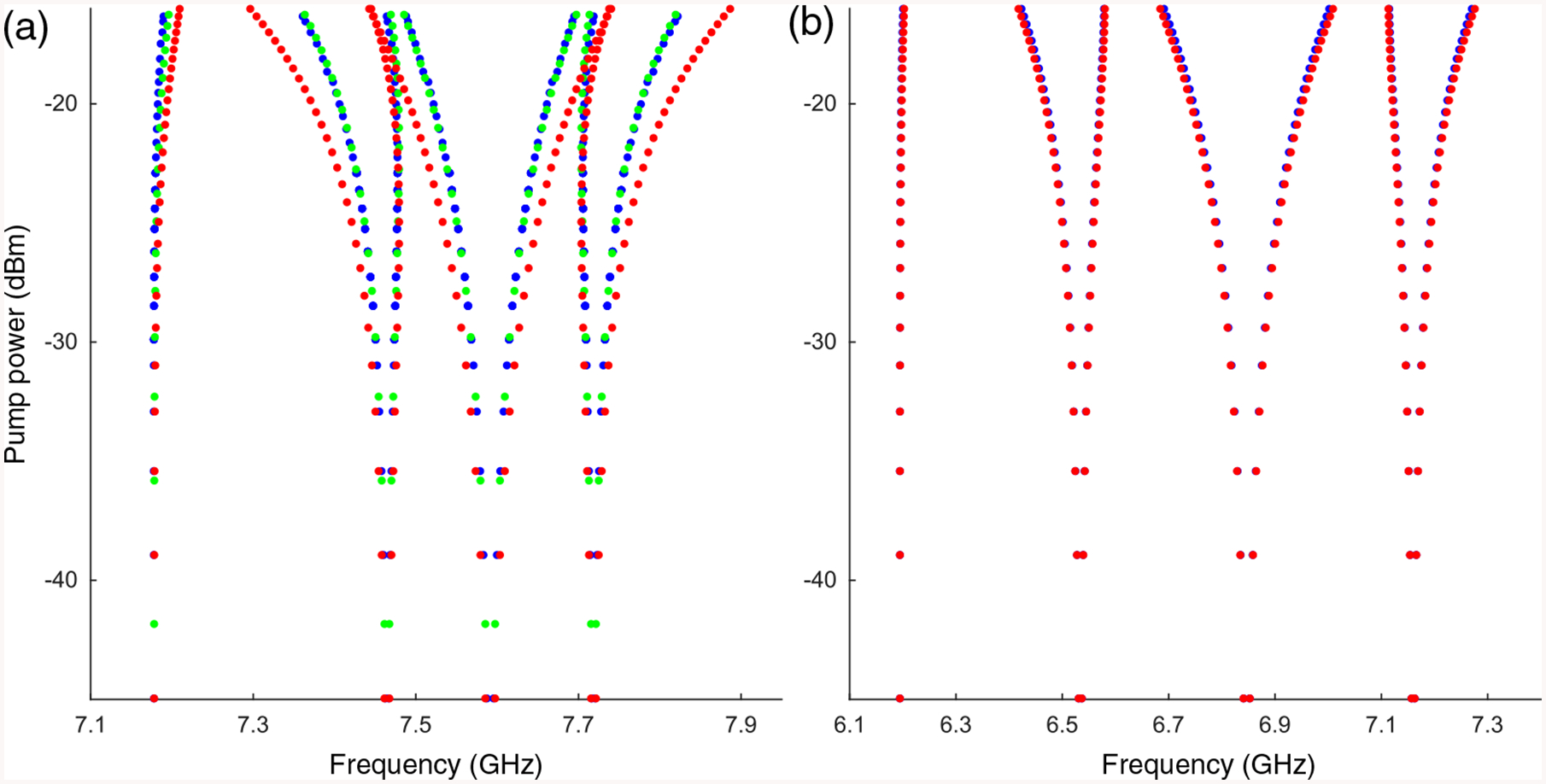
Theoretical simulations of the emission spectrum for different pump powers. Here, the blue squares are numerical results obtained from the total Hamiltonian Eq. (E1), the green squares are from the dressed-transmon Hamiltonian Eq. (E2), and the red squares are from Eq. (E2) but with cos *θ* = 1. (a) Here, *ω*_01_ = 7.97 GHz, and the bound-state frequency is 7.591 GHz. Using the appropriately reduced matrix element (cos *θ* < 1, blue squares) is crucial in obtaining accurate results using Eq. (E2). (b) Here, the bare qubit frequency of 7 GHz and the bound-state frequency of 6.847 GHz are both far from the band edge, so that cos *θ* ≈ 1 and the reduction in the matrix element can be neglected, so all three data sets lie on top of each other.

**FIG. 10.**
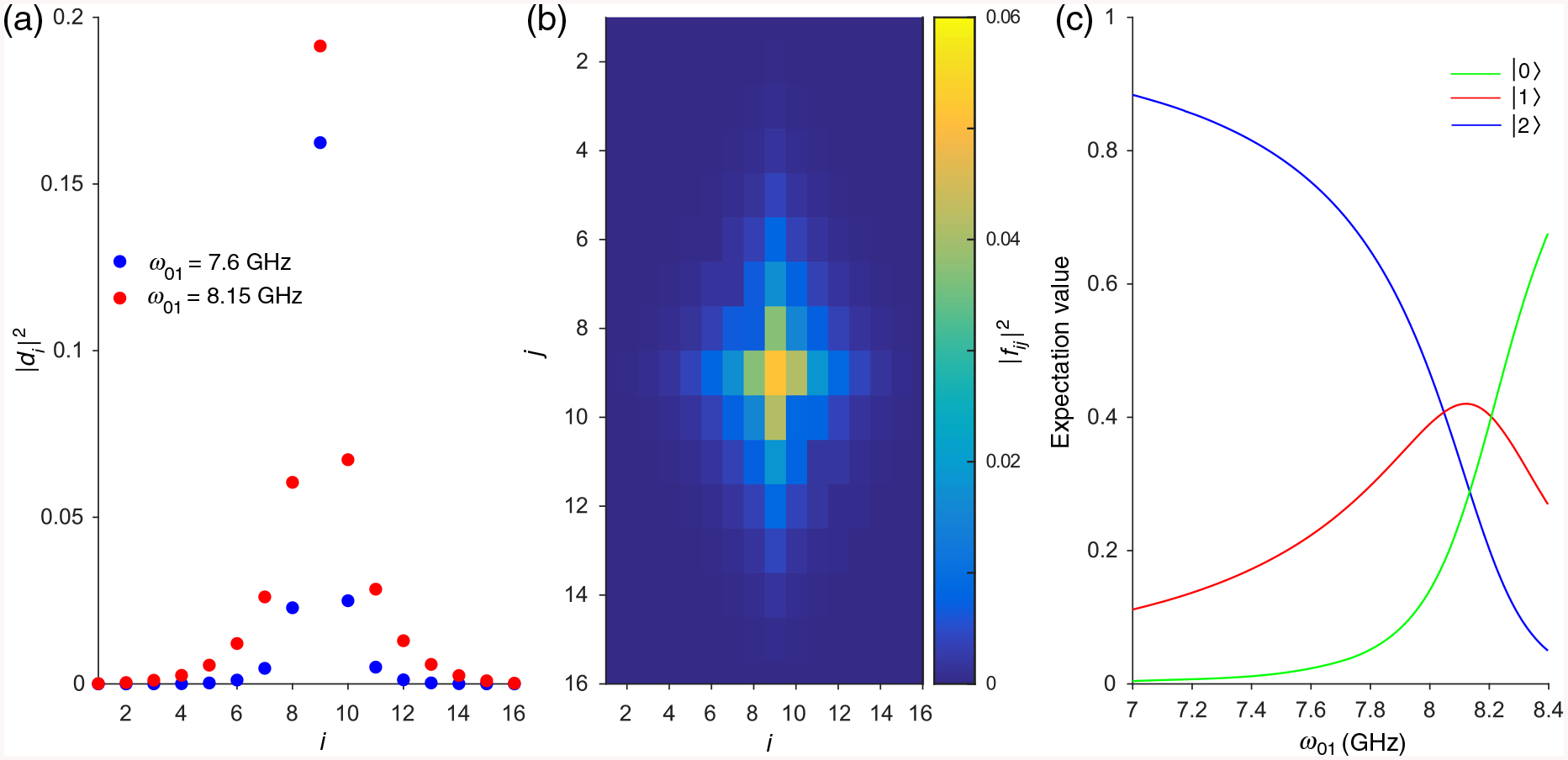
Visualizing the two-photon bound state. Here, the transmon qubit is on site nine. (a) The blue dots are |*di|*^2^ versus position for *ω*_01_ = 7.6 GHz. The red dots are |*di|*^2^ for *ω*_01_ = 8.15 GHz as a function of position. (b) Plot of |*f*_*i*,*j*_*|*^2^ as a function of *i* and *j* for *ω*_01_ = 8.15 GHz. (c) The population of transmon qubit levels in the two-excitation ground state of *H*_tot_ as a function of bare qubit frequency.

## APPENDIX A: MOTIVATION FOR USING PHOTONIC CRYSTALS

In this Appendix, we discuss our motivation for using photonic crystals and their advantages over other devices that might realize bound states. To realize a dressed bound state between a qubit and a band edge, we must couple a qubit to a photonic band edge, where a band edge consists of photonic states corresponding to the transition between a stop band and a passband that are “slow light” due to reduced group velocity. Band gaps and passbands are not unique to photonic crystals—we come across them in numerous other structures such as waveguides (near cutoff frequency) or aperiodic filters.

To motivate the benefit of photonic crystals, let us consider using an aperiodic filter instead. Filters are ubiquitous in the microwave domain with many established design methods that trade off optimizing various parameters such as roll-off or passband ripple. Stepped impedance filters are a standard model for implementing filters, where each impedance step is chosen precisely to meet filter design constraints with no periodicity requirement [30]. Because of this, aperiodic filters may also be more sensitive to fabrication errors than periodic filters.

The next requirement is to couple a qubit. However, the lack of periodicity transfers also to the electric field distribution (no Bloch modes), and so we must numerically calculate the optimal location to place the qubit and recalculate for every modification of filter design. While this seems feasible for a single qubit, the lack of Bloch modes is highly problematic from a scalability standpoint as it is not guaranteed that we can couple multiple qubits (or even just two), with near identical coupling strengths, to that band edge.

Furthermore, from a theoretical perspective, periodic crystals are simpler, cleaner structures that are described in the infinite limit by dispersion relations in momentum space, providing useful insight for predicting system behavior. Therefore, while it may be possible to realize dressed bound states with a qubit in an aperiodic structure, the benefits from using a periodic structure far outweigh the likely larger device footprint.

A photonic crystal is an electromagnetic structure formed by periodic modulation of the dielectric constant. This results in dispersion relations characterized by energy bands—alternating band gaps (“forbidden energies”) and passbands (continuous density of states). The electric field distribution is characterized by Bloch modes, allowing for the position of qubits at locations that optimize coupling to the band edge [8]. Engineering band edges via finite-size photonic crystals has been demonstrated in many systems, including nanophotonic structures [47,48] and superconducting microwave coplanar waveguides [13,49], and tremendous progress towards integration with ultracold atoms and superconducting qubits, respectively, has been reported [12,13]. There are other ways to create superconducting microwave crystals, including using Josephson junction arrays or lumped element circuits.

Our approach to creating an effective 1D microwave photonic crystal is periodically alternating sections of high and low impedance coplanar waveguides (CPWs). With CPWs, the impedance can be easily changed via the center-pin width and the center-pin to ground plane (the gap) distance.

We define a unit cell as a high impedance section of length *L*_hi_ and impedance *Z*_hi_ sandwiched between two sections of low impedance of lengths *L*_lo_/2 and impedances *Z*_lo_ (for symmetry purposes). With a periodic modulation, there are naturally many gaps in the band structure. We chose to more strongly couple the qubit to the second band edge, rather than the first, because it has a smoother passband while still having a steep roll-off.

### 1. Crystal simulation and parameters for implementation

We can use the unit cell to calculate the band structure or dispersion relation for an infinite crystal. While we can never make an infinite crystal, calculating the dispersion relation is a very useful starting point and gives us insight into crystal parameters such as band curvature. To a good approximation, the phase velocities in the high and low impedance CPW sections are effectively equal (*v*_*p*;high_ ≈ *v*_*p*;low_ ≈ *v*_*p*_ ≈ 1.248 × 10^8^ m/s). This yields

(A1) $$\text{cos}\left(\frac{\omega_{k}L_{\text{Io}}}{v_{p}}\right)\text{ cos}\left(\frac{\omega_{k}L_{\text{hi}}}{v_{p}}\right)\;-\frac{1}{2}\left(\frac{Z_{\text{hi}}}{Z_{\text{Io}}}+\frac{Z_{\text{Io}}}{Z_{\text{hi}}}\right)\text{ sin}\left(\frac{\omega_{k}L_{\text{Io}}}{v_{p}}\right)\text{ sin}\left(\frac{\omega_{k}L_{\text{hi}}}{v_{p}}\right)\;=\text{cos}\left[k\left(L_{\text{Io}}+L_{\text{hi}}\right)\right],$$

where *k* is the momentum and *ω*_*k*_ is the dispersion.

We determine the band structure dependence on *L*_lo_ and *L*_hi_ for *Z*_lo_ ≈ 25 Ω and *Z*_hi_ ≈ 125 Ω. We look to optimizing the trade-offs across four parameters: the frequency of the band edge, the width of the band gap, the width of the passband, and the curvature of the band. From these simulations, we see that small changes in unit cell impedances do not lead to significant changes in the band dispersion.

For comparison, Figs. 5(a) and 5(b) show the simulated dispersion for unit cell parameters in Liu *et al*. [13] and the present paper, respectively. The unit cell for the present paper was chosen to have a flatter band dispersion (analogous to effective mass), *α*, so as to realize more localized bound states.

While the dispersion relation assumes an infinite system, crystals of small, finite length have been shown to realize well-resolved gaps in dispersion where transmission is suppressed and bands where transmission is unimpeded. From an experimental perspective, we use transmission-based measurements to probe the states.

Transfer matrices are a convenient and accurate method for bare crystal simulation that incorporates both the exact number of cells and boundary conditions [31]. A convenient metric for comparison is transmission across the device, *S*_21_. In Figs. 5(c) and 5(d), comparison of the measured *S*_21_ of the bare crystal (taken at powers high enough to saturate qubit effects) and the calculated *S*_21_ from transfer matrices shows agreement [13].

### 2. Realistic parameters and experimental aside

The device is fabricated on an approximately 430-*μ*m-thick *c*-plane sapphire substrate, on which we sputter approximately 200 nm of niobium. The photonic crystal is patterned using a direct-write laser writer, followed by a dry *SF*_6_ reactive ion etch to transfer the pattern. To ensure our waveguides are reasonably sized and can be reliably fabricated using photolithography, we are limited to impedances between approximately 25 and 125 Ω.

For this device, one unit cell consists of a high impedance section (*Z*_hi_ = 124 Ω, *L*_hi_ = 7.8 mm) and a low impedance section (*Z*_lo_ = 25 Ω, *L*_lo_=1.2mm). Impedance estimates are obtained by fitting the measured spectrum with transfer matrix simulations; the dispersion is robust to small impedance deviations in the fabricated sample. We fit 16 unit cells on a 10 mm × 10 mm sapphire chip. While more unit cells could fit on the chip, we see experimentally that we must wire-bond extensively on-chip (to connect ground planes) to create clean band gaps and passbands and included this requirement into the design. By switching to air or dielectric-supported bridges in the future we would be able to shrink the device footprint or include more unit cells in the same-size device.

To integrate with the standard measurement setup and qubit parameters, we choose unit cell lengths such that the second band edge falls between 7 and 8 GHz. The frequency of the band edge was designed to be at 7.8 GHz to match with the traveling-wave parametric amplifier (TWPA, dispersion feature around 8.3 GHz) to provide good amplification for frequencies in the vicinity of the band edge as well as deep in the band gap. The width of the band gap is from 4.75 to 7.8 GHz and needs to be large enough such that we have a wide range to tune the qubit frequency over, which would in turn result in a wide range of accessible localization lengths. The width of the (second) passband needs to be sufficiently large such that we can ignore the third band edge and we can make the approximation that the curvature of the band (near the band edge) is quadratic. Finally, as the curvature of the band plays a role in determining the localization length, to make the states more localized, we chose a shallower band curvature [compare red plots in Figs. 5(a) and 5(b)].

In future iterations, one may consider altering boundary conditions specific to the desired application. For example, methods for impedance matching such as tapers or quarter-wave transformers [30] are straightforward additions that will modify impedance matching at specific frequencies (such as at a band edge). These options were not pursued in this work as we wanted to study bound-state properties across a range of frequencies. If one were interested in only specific frequencies or ranges, then this is a promising improvement. The device is symmetric, so one end is chosen arbitrarily to serve as the input. As detection of the bound state is due to scattering, one may consider modifying the symmetry of the device or detecting signal from both ends of the crystal to improve collection efficiency.

We resort to two-tone spectroscopy over direct transmission measurement to detect the bound state when the qubit is deep in the band gap due to strong localization and poor signal-to-noise ratio (SNR). Choosing a less shallow band curvature will make it possible to see the bound state all the way through the gap (see Liu *et al*. [13]); however, this is a trade-off as the bound-state linewidths will increase accordingly. Thus, choosing curvature and crystal parameters such that the linewidth remains as narrow as possible without internal Q or loss effects is essential.

## APPENDIX B: ADDING IN QUBITS

In this Appendix, we discuss the experimental details of coupling the transmons to the photonic crystal. In this device, we capacitively couple a transmon qubit to each of the two central unit cells of the 16-cell crystal. A transmon’s anharmonic level structure is due to the nonlinear inductance of Josephson junctions and allows for selective addressing of energy level transitions. However, it is important to emphasize that our realization of a qubit does have higher energy levels set by transmon geometry, unlike the standard theoretical qubit which is synonymous with a two-level system. These higher levels are also coupled to the band edge and therefore accounting for these levels becomes important when looking beyond the first excitation sector.

Our qubits are fully patterned with a 125-kV *e*-beam writer, with bridge-free junctions, and are made of evaporated aluminum. The qubits are designed to have a target charging energy (from electromagnetic simulation) of approximately 450 MHz, and emphasis is placed on maximizing coupling between the qubit and photonic crystal without significantly altering the unit cell. Finally, although identical in design, in reality the qubits will differ due to fabrication variability. However, as coupling to the waveguide is designed to be the dominant decay channel for the qubits, bound states are robust to small variation in other parameters.

We place qubits in adjacent unit cells (9-mm separation) at the center of the crystal such that we can see significant change in interaction strength with detuning. Each qubit has a local flux bias line for independent control—DC cross talk is corrected for through standard calibration. These lines are low pass filtered; however, as the dominant decay of the qubit is via the crystal, this is not expected to be a limiting factor in coherence.

To determine where to place the qubits within the unit cell such that they maximally couple to the desired band, we must calculate the electric field distribution within the unit cell, distribution determined by the Bloch modes for the crystal [8].

For these crystal parameters, maximally coupling to the second band edge (and minimally to the first band edge) corresponds to placing the qubit in the center of long high-impedance section. Other locations within a unit cell change coupling to each band edge, which is a potentially interesting regime for future experiments. However, here we are interested in reducing the effect of the other band as much as possible. We cannot completely eliminate this coupling—experimentally we can still detect a drop in transmission in the lower passband when the qubit is resonant but this change is orders of magnitude smaller than in the second band.

## APPENDIX C: MODEL, TRANSMISSION, AND FITTING OF PARAMETERS

In this Appendix, we introduce the effective Hamiltonian for our system and discuss the fitting of various parameters of the Hamiltonian in detail.

### 1. Hamiltonian

The Hamiltonian for the one-dimensional photonic crystal is given by $H_{c}=\sum_{k}\omega_{k}a_{k}^{†}a_{k}$, where $a_{k}^{†}$ creates a bosonic excitation with momentum *k*, and *ω*_*k*_ is the dispersion relation of the second band of the photonic crystal, which is discussed in Sec. A 1. Here, we ignore the other bands of the photonic crystal as the qubits couple predominantly to the second band. Fourier transforming $(a_{k}^{†}=\sqrt{a/L}\sum_{j=1}^{N}a_{j}^{†}e^{ikx_{j}}$, where *L* is the system size, *a* is the unit cell size, *N* is the number of unit cells, *x*_*j*_ = *aj*, and *k* = [(2*π*)/*L*]*n*, where *n* is an integer) gives a hopping model with periodic boundary conditions, $H_{c}=\sum_{i,j}J_{i,j}a_{i}^{†}a_{j}$, where

(C1) $$J_{i,j}=J_{\left|i-j\right|}=\underset{N\to \infty }{\text{lim}}\frac{1}{N}\sum_{k}e^{ik\left(x_{i}-x_{j}\right)}\omega k\;=\;\int_{-\pi }^{\pi }\frac{d\tilde{k}}{2\pi }\;e^{i\tilde{k}\left(x_{i}-x_{j}\right)/a}\omega_{\tilde{k}/a}.$$

Here, $\tilde{k}$ is a dimensionless integration variable, and we use the fact that *J*_*i*,*j*_ = *J*_*j*,*i*_ since *ω*_*k*_ = *ω*_−*k*_. In Eq. (C1) we take *N* → ∞, as we only know *ω*_*k*_ in that limit (see Sec. A 1). To model our finite system, which has open boundary conditions and 16 unit cells, we use a 16-site hopping model with open boundary conditions with hopping strengths determined by Eq. (C1). Using the photonic crystal parameters in Sec. A 2, we find (by numerical integration) *J*_0_ = 9.3272 GHz, *J*_1_ = 0.7288 GHz, *J*_2_ = −0.0344 GHz, *J*_3_=0.0178GHz, *J*_4_= −0.0034GHz, and *J*_5_=0.0014GHz. Unfortunately, we are unaware of an exact analytical solution for *J*_*i*,*j*_ for our system. In our numerical simulations, we keep hopping terms up to *J*_5_. We calculate the hopping parameters for a given set of photonic crystal parameters. A different choice of photonic crystal parameters would have given a different set of hopping parameters. As such, these parameters should only be understood as estimates. We briefly comment on the change in theory parameters that arises from using different photonic crystal parameters at the end of this section.

The Hamiltonian for the isolated transmon qubits is given by

(C2) $$H_{q}=\sum_{i=1,2}\sum_{n=0}^{\infty }\omega_{0n;i}{\left|{n\rangle }_{i}\langle n\right|}_{i}.$$

Here, *i* labels the transmon qubit, *n* labels the level of the transmon qubit and *ω*_0*n*;*i*_ are the bare energy levels of the transmon qubits. In our simulation, the number of transmon qubit levels is truncated at five (i.e., |0〉 through |4〉). For our experiment, *ω*_00;*i*_ =0, *ω*_02;*i*_=2*ω*_01;*i*_−Δ_*i*_, *ω*_03;*i*_=3*ω*_01;*i*_−3Δ_*i*_, and *ω*_04;*i*_ = 4*ω*_01;*i*_ − 6Δ_*i*_, where Δ_*i*_ is the bare anharmonicity of the *i*th transmon qubit.

We now turn to the coupling of the transmon qubits to the photonic crystal. To an excellent approximation, the coupling between the transmon qubit and the photonic crystal takes place within a single unit cell (see Appendix D 2 for justification of this statement). Thus, in the rotating-wave approximation, we can write the coupling term of the Hamiltonian as

(C3) $$H_{qc}=\sum_{i=1,2}g_{i}a_{z_{i}}^{†}\left({|0\rangle }_{i}{\langle {1|}_{i}+\sqrt{2}|1\rangle }_{i}{\langle {2|}_{i}+\sqrt{3}|2\rangle }_{i}\;\times {\langle {3|}_{i}+\sqrt{4}|3\rangle }_{i}\langle {4|}_{i}\right)\;+\text{ H.c.},$$

where *z*_*i*_ labels the position of the two transmon qubits. In our system, the transmon qubits are on neighboring unit cells, i.e., *z*_1_ = (N/2) and *z*_2_ = (N/2) + 1, and the coupling for each transmon qubit, *g*_*i*_, is different due to small experimental imperfections. The total Hamiltonian of the system is then

(C4) $$H_{\text{tot}}=H_{c}+H_{q}+H_{qc}.$$

Finally, we note that this Hamiltonian conserves total excitation number.

### 2. Transmission methods

We now discuss the two theoretical methods we use to calculate transmission in the linear drive regime. Neither method relies on a weak coupling approximation between the transmon qubits and the photonic crystal. The first method involves treating the system as an open quantum system, with loss on each site (that is site dependent), subject to a weak drive on the first site. The largest loss terms are at the ends of the one-dimensional photonic crystal. The system can then be described by the following effective non-Hermitian Hamiltonian (in the rotating frame) with driving frequency *ω*_*d*_ and strength *ϵ*,

(C5) $$H_{\text{eff}}=\sum_{i,j=1}^{N}\left(J_{i,j}-\omega_{d}\delta_{i,j}-i\kappa_{0}\delta_{i,j}\right)a_{i}^{†}a_{j}\;+\sum_{i=1,2}\left(\omega_{01;i}-\omega_{d}-i\kappa_{q}\right){\left|{1\rangle }_{i}\langle 1\right|}_{i}\;+\sum_{i=1,2}g_{i}\left(a_{z_{i}}^{†}{|0\rangle }_{i}{\langle 1\left|{}_{i}+\;a_{z_{i}}\right|1\rangle }_{i}\langle 0|{}_{i}\right)-i\kappa \left(a_{1}^{†}a_{1}+a_{N}^{†}a_{N}\right)\;+\epsilon \left(a_{1}^{†}+a_{1}\right),$$

where *κ*_*q*_ is the qubit half-width, *κ*_0_ is a uniform contribution to photonic half-width, and *κ* is a decay rate on the first and last sites. While there are certainly other forms of loss (such as nonuniform loss on each site), our goal is to reproduce the key features (e.g., the locations of the bound state and of the transmission dip, as well as the linewidth of the bound state) using as few parameters as possible. The equations of motion for the quantum operators are

(C6) $$\frac{\partial a_{y}}{\partial t}=-i\left(\sum_{j}\left(J_{y,j}-\omega_{d}\delta_{y,j}\right)a_{j}+\epsilon \delta_{y,1}+\sum_{i=1,2}\delta_{y,z_{i}}g_{i}{\left|{0\rangle }_{i}\langle 1\right|}_{i}\right)\;-\left[\kappa_{0}+\kappa \left(\delta_{y,1}+\delta_{y,N}\right)\right]a_{y},$$

(C7) $$\frac{\partial \left({\left|{0\rangle }_{i}\langle 1\right|}_{i}\right)}{\partial t}=\left[-i\left(\omega_{01;i}-\omega_{d}\right)-\kappa_{q}\right]|0\rangle_{i}\;\times \langle 1|{}_{i}-ig_{i}a_{z_{i}}\left({|0\rangle }_{i}{\langle 0\left|{}_{i}-\;\right|1\rangle }_{i}\langle 1|{}_{i}\right).$$

We omit vacuum Langevin noise from the equations of motion as this noise does not affect our calculations. Solving for the steady state of *a*_*N*_ in the weak drive limit (〈|1〉_*i*_〈1|*i*〉*ss* = 0) yields the transmission. More specifically, *S*_21_ ∝ 〈*a*_*N*_〉_*ss*_*/ϵ*.

The second method we use was introduced by Biondi *et al*. [29]. Here, we treat the system (which is taken to be the photonic crystal and qubits) as being connected to waveguides with linear dispersions, with velocity *v*_*g*_, at the ends of the photonic crystal. In this method, we take *κ*_*q*_ = *κ*_0_ = *κ* = 0, so that the single-excitation transmission through the system can be expressed in terms of eigenvalues and eigenvectors of the system described by *H*_tot_ [Eq. (C4)] [29]. More explicitly, the transmission for a given frequency ω is given by |*T*(*ω*)|^2^, where

(C8) $$T(w)=\frac{-2i\beta }{\Gamma_{l}\Gamma_{r}+|\beta {|}^{2}},\;\Gamma_{l,r}=1+\frac{i}{2v_{g}}\sum_{n}\frac{{\left|V_{n}^{l,r}\right|}^{2}}{\omega -\Omega_{n}},\;\beta =\frac{1}{2v_{g}}\sum_{n}\frac{V_{n}^{l}{\left(V_{n}^{r}\right)}^{*}}{\omega -\Omega_{n}}.$$

Here, $V_{n}^{l,r}=\sqrt{v_{g}g_{w}}\langle 0\left|a_{1,N}\right|n\rangle$, Ω*n* and |*n*〉 are the eigenvalues and eigenvectors of *H*_tot_ in the single-excitation sector, and *g*_*w*_ is the coupling between the waveguide and the photonic crystal. Intuitively, transmission occurs when the single-excitation eigenstates have the probability of the photon being on both ends of the photonic crystal. Near the bound-state energy *E*_1*B*_, we can write the transmission as

(C9) $$|T(\omega ){|}^{2}\approx \frac{1}{4v_{g}^{2}}\frac{V^{4}}{{\left(\omega -E_{1B}\right)}^{2}+\frac{1}{v_{g}^{2}}V^{4}}.$$

Here, we have assumed that the wave function is symmetric, i.e., $V_{n}^{l}=V_{n}^{r}=V$. We see that the linewidth (in the limit) is proportional to the wave function at the edge of the system *V*, as expected.

### 3. Fitting of parameters

In this section, we discuss how we fit various parameters of the total Hamiltonian. The unknown parameters include *g*_*i*_ and Δ, and, for the first method, also *κ*_0_, *κ*_q_, and *κ*. Furthermore, *ω*_01;*i*_ is tunable but its value is unknown, and the transmission dip (visible when the bare qubit frequency is in the passband) does not, in general, occur at the bare qubit frequency unless hopping amplitudes *J*_*i*,*j*_ beyond nearest neighbor are negligible.

The first parameters we determine are *κ* and *κ*_0_ (using the first transmission method). We turn off the coupling of the qubits to the photonic crystal (in the experiment, this is accomplished by saturating the qubits). We set *κ*_0_ and *κ*_*q*_ to zero and fit κ. Given that the largest losses occur at the ends of the photonic crystal, fitting *κ* first is reasonable. *κ* controls the linewidth of the photonic modes and the transmission amplitude difference between the transmission dips and peaks in the passband. We find that a reasonable estimate for *κ* [for the experimental data in Fig. 5(d)] is 1 GHz, although any *κ* in the range of 0.5 to 1.5 GHz also gives a reasonable fit. We then turn on *κ*_0_, which further reduces the transmission amplitude difference between the transmission dips and peaks in the passband and lowers the transmission peak of the lowest photonic mode. We estimate that *κ*_0_ = 4 MHz (although any *κ* in the range of 3 to 5 MHz also fits the data well). Figure 5(d) shows that simulated transmission is in good agreement with the experimental data. Given that there are other losses in the system that we have not included, these numbers should be understood as estimates.

We now turn to determining *g*_*i*_. While *κ*_*q*_ is set to zero for now, we find that varying it or the other loss parameters does not noticeably change the frequency of the bound state or transmission dip, thus making our estimate of the coupling strength independent of the decay parameters. To begin, we detune the qubit at site *N/*2 far away from the passband (in the experiment, the detuned qubit is at 4.5 GHz) and then fix the other (i.e., second) bare qubit frequency [50]. Transmission is then calculated as a function of driving frequency. We find that *g*_2_ = 0.55 GHz and *ω*_01;2_ = 7.9875 GHz match the experimental data well when the bound state is at 7.605 GHz, as seen in Fig. 6(a). Calculating transmission when the first qubit frequency is near the passband and the second qubit frequency is detuned and comparing it to experimental data, we find *g*_1_ = 0.505 GHz [50]. To make sure we have chosen suitable coupling strengths, we tune the bare qubit frequency (the detuned bare qubit frequency is kept fixed). If we have chosen the correct parameters, we should accurately capture the locations of the bound state and the transmission dip for different bare qubit frequencies, while keeping the other parameters fixed. Indeed, as seen in Fig. 6(b), we find this is the case for the chosen coupling strengths.

Our next goal is to obtain an estimate for *κ*_*q*_. To do so, we increase *κ*_*q*_, which increases the linewidth of both the transmission dip and the bound state, until the linewidth of the bound state matches the experimental value well for a fixed bare qubit frequency (we note increasing *κ*_0_ also increases the linewidth of the bound state, however *κ*_0_ is already fixed). We find that *κ*_*q*_ = 0.5 MHz accomplishes this task for *ω*_01;2_ = 7.9875 GHz. To make sure we have chosen a suitable qubit half-width, we again tune the second bare qubit frequency (while keeping the detuned bare qubit frequency fixed). If we have chosen a reasonable qubit half-width, we should be able to accurately estimate the linewidth of the bound state for different bare qubit frequencies (while keeping all other parameters fixed). Figure 1(b) of the main text shows that our estimate of *κ*_q_ is reasonable.

Before moving on, we briefly comment on the second transmission method. Figure 6(c) shows theoretical data from both simulations for *g*_2_ = 0.55 GHz and *ω*_01;2_ = 7.9875 GHz. The locations of the bound state and transmission dip occur at the same spot for both methods. The key difference is that the second method does not accurately predict the magnitude of the linewidth of the bound state as it assumes *κ*_*q*_ = *κ*_0_ = *κ* = 0 (but instead has coherent coupling of the crystal to the waveguide). In these simulations, we have taken *g*_*w*_ = 2 GHz, as that fits the data when the qubits are saturated well (not shown). We note that any value of *g*_*w*_ in the range of 1.5 to 2.5 GHz also gives a reasonable fit to the saturated qubit data and that the frequencies of the bound state and the transmission dip are not sensitive to *g*_*w*_. Simulated transmission data presented in the main text is from method one.

We now fit the last remaining parameters, Δ_1_ and Δ_2_, which are the bare transmon anharmonicities. We first diagonalize *H*_tot_ in the two-excitation sector for fixed *ω*_01;*i*_ (with the qubit on site *N*/2 detuned far away). The theoretical prediction for the dressed anharmonicity of the qubit on site *N*/2 + 1 is found by taking the lowest eigenvalue of *H*_tot_ in the two-excitation sector and subtracting two times the lowest single-excitation eigenvalue. We vary Δ_2_ until the theoretically predicted dressed anharmonicity of the second qubit matches the experimentally measured dressed anharmonicity for a given bare qubit frequency (we choose our bare qubit frequency such that the single-particle bound state is at 7 GHz). In doing so, we find, to a good approximation, that Δ_2_ = 0.365 GHz. We then vary the bare qubit frequency and make sure the theoretically predicted dressed anharmonicity is still consistent with the experimentally measured value for different bare qubit frequencies. We find excellent agreement for a wide range of bare qubit values as shown in Figs. 2(a) and 2(b) in the main text. Finally, we choose the value of Δ_1_ to give the best fit to the magnitude of the two-photon avoided crossing [see inset of Fig. 4(a) in the main text]. In doing so, we find that Δ_1_ = 0.365 GHz fits the experimental data reasonably well.

Before we close this section, we estimate errors in our parameters. To begin, we estimate what change in hopping parameters (we call these different hopping parameters *J*′) we would get if we choose *Z*_high_ = 123.5 Ω instead of 124 Ω. Both of these choices fit the experimental data well in transmission matrix simulations. This choice of *Z*_high_ gives *J*′_0_=9.331GHz, *J*′_1_ =0.7308GHz, *J*′_2_ − .0345GHz, J′_3_=0179 J′_4_=−0035GHz, and *J*′_5_=0.0014GHz. We see that the ratio of these hopping parameters to the previous set is not less than 0.97 for any term, so we expect the other parameters in our model will not differ by more than 5% from their given predictions. We also expect this to hold true if one uses any reasonable set of photonic crystal parameters. To test this, we estimate *g*_2_ and the range of decay parameters for the second set of hopping parameters. We find *g*_2_ = 0.555 GHz and that the same range of decay parameters fit the data well, consistent with our expectation.

## APPENDIX D: BOUND-STATE FUNDAMENTALS

In this Appendix, we discuss the theory of bound states. Strong light-matter interaction between atoms and slow-light structures, ones with vanishing or significantly reduced group velocity such as photonic band edges in photonic crystals, has been an area of ongoing interest both in theory and recent experiment. The principal interest behind this study is the localized *bound* photonic state that forms around the atom. This bound state has an exponentially decaying photonic envelope that tunes with detuning of the qubit transition from the band edge (see Fig. 7). While the bound state is always within the gap, the frequency of the bound state changes with qubit frequency [see Figs. 1(e)–1(f)]. Additionally, the state becomes less localized as the bare qubit is tuned closer to the band edge [see Figs. 7(a) and 7(b)].

In a finite-size system, these localized states overlap with the ends of the crystal, thus facilitating single-photon transport across the crystal at the bound-state frequency and providing an avenue to probe these states. This tunable photonic interaction mechanism provides a platform for simulation of many-body quantum optics in one-dimensional systems, distinct from cavity or waveguide quantum electrodynamics [see Figs. 7(c) and 7(d)].

In Figs. 1(e) and 1(f), we measure (simulate) *S*_21_ at low power to track the bound state as a function of qubit frequency. The bound state shows up as a Lorentzian peak in transmission. We detect the change in wave-function overlap as a change in bound-state linewidth—where linewidth increases with localization length [see Fig. 1(c) in [Ref. 13]]. As discussed in Sec. A, the localization of the photonic wave function is determined predominantly by the strength of the coupling, properties of the band edge, and the frequency detuning between the atom and the band edge. The ability to tune the localization with small-sized crystals shows the versatility of this platform.

### 1. Bound-state linewidth dependence on detuning

The linewidth of the bound state is set by the amplitude of the exponentially decaying photonic wave function at the ends of the crystal (ignoring other forms of loss). The envelope amplitude decays as ~*e*^−*x/L*^, where *x* is the distance from the qubit location and *L* is the localization length. For simplicity, we consider the case of a single qubit at the center of the crystal (total length *d*) such that the envelope is symmetric. This results in a linewidth proportional to *e*^−*d*/2*L*^. Of course, this approximation is only valid in the limit where *e*^−*d*/2*L*^ ≪ 1, meaning the bound state is sufficiently localized compared to the finite length of the crystal.

### 2. Single-photon bound states: Exact solution

In this section, we discuss the theory of atom-photon bound states in the single-excitation sector for an infinite photonic crystal coupled to two qubits. While our system is of course finite, these results provide an intuitive understanding of our system. We note that a similar calculation for two qubits was first done, to the best of our knowledge, in Ref. [6] and then later in Ref. [7] for the case when the two qubits have equal coupling strengths and equal qubit frequencies. The results below generalize that work to unequal coupling strengths and unequal qubit frequencies. To begin, we first write the Fourier transformed Hamiltonian from a more microscopic point of view (ignoring decay and ignoring terms that do not affect the single-excitation bound states),

(D1) $$H=\sum_{k}\omega_{k}a_{k}^{†}a_{k}+\;\sum_{i=1,2}\omega_{01;i}{|1\rangle }_{i}\langle {1|}_{i}+\frac{1}{\sqrt{N}}\sum_{i=1,2}\left(\sum_{k}g_{k,i}\left(a_{k}^{†}|{0\rangle }_{i}{\langle 1\left|{}_{i}+g_{k,i}^{*}a_{k}\right|1\rangle }_{i}\langle {0|}_{i}\right)\right).$$

To make analytical progress, we assume that the dispersion relation is $\omega_{k}=\omega_{0}+\alpha a^{2}{\left[k\mp (\pi /a)\right]}^{2}$ which is valid around *k =* ±*π*/*a*. While we chose a quadratic dispersion, these results are qualitatively similar for a cosine dispersion. Here, we also restore the momentum dependence of the coupling strength $g_{k,i}=\sqrt{\omega_{k}/2\hbar }d_{i}u_{k}\left(r_{i}\right)$, where *d*_*i*_ is the matrix element between |0〉 and |1〉 states for the *i*th qubit and *u*_*k*_(*r*_*i*_) is the spatial function for the *k*th mode evaluated at the *i*th qubit position, *r*_*i*_ (see Ref. [6]). As *ω*_0_ is much larger than the bandwidth, we can approximate $\sqrt{\omega_{k}}$ as $\sqrt{\omega_{0}}$. Furthermore, for our system, *u*_*k*_(*r*_*i*_) for the second band is nearly independent of momentum as shown in Ref. [13]. Thus, to an excellent approximation, *g*_*k*,*i*_ is constant, which we define as *g*_*k*,*i*_ = *g*_*i*_, and hence the coupling is local in real space. Finally, we note that *u*_*k*_(*r*_*i*_) for the first band vanishes when the qubit is in the middle of the high-impedance section, thus we can ignore coupling to the first band to an excellent approximation. The most general single-excitation wave function is

(D2) $$|\psi_{1B}\rangle =\text{sin }\phi \left(a_{1}|1\rangle_{1}|0\rangle_{2}|0\rangle +a_{2}|0\rangle_{1}|1\rangle_{2}|0\rangle \right)\;+\text{cos }\phi \sum_{k}c_{k}a_{k}^{†}|0\rangle_{1}|0\rangle_{2}|0\rangle .$$

Here, the basis states are labeled as |qubit one〉_1_|qubit two〉_2_|photon〉, and the conditions, $a_{1}^{2}+a_{2}^{2}=1$ and $\sum_{k}{\left|c_{k}\right|}^{2}=1$, ensure the wave function is properly normalized. Solving the eigenvalue equation *H*|*ψ*_1*B*_〉 = *E*_1*B*_|*ψ*_1*B*_〉 yields the following coupled equations:

(D3) $$\text{sin }\phi E_{1B}a_{1}=\text{sin }\phi \omega_{01;1}a_{1}+\text{cos }\phi \frac{g_{1}}{\sqrt{N}}\sum_{k}c_{k}e^{-ikaz_{1}},$$

(D4) $$\text{sin }\phi E_{1B}a_{2}=\text{sin }\phi \omega_{01;2}a_{2}+\text{cos }\phi \frac{g_{2}}{\sqrt{N}}\sum_{k}c_{k}e^{-ikaz_{2}},$$

(D5) $$\text{cos }\phi E_{1B}c_{k}=\text{cos }\phi \omega_{k}c_{k}+\text{sin }\phi a_{1}\frac{g_{1}}{\sqrt{N}}e^{ikaz_{1}}\;+\text{sin }\phi a_{2}\frac{g_{2}}{\sqrt{N}}e^{ikaz_{2}}.$$

Solving for *c*_*k*_ from Eq. (D15) and inserting the result into Eq. (D3) yields

(D6) $$E_{1B}a_{1}=\omega_{01;1}a_{1}+a_{1}\frac{g_{1}^{2}}{N}\sum_{k}\frac{1}{E_{1B}-\omega_{k}}\;+a_{2}\frac{g_{1}g_{2}}{N}\sum_{k}\frac{e^{-ika\left(z_{1}-z_{2}\right)}}{E_{1B}-\omega_{k}}.$$

The sums are evaluated as follows:

(D7) $$\frac{1}{N}\sum_{k}\frac{1}{E_{1B}-\omega_{k}}=a\int_{0}^{\pi /a}\frac{dk}{2\pi }\frac{1}{E_{1B}-\omega_{0}-\alpha a^{2}{\left(k-\frac{\pi }{a}\right)}^{2}}\;+\;a\int_{-\pi /a}^{0}\frac{dk}{2\pi }\frac{1}{E_{1B}-\omega_{0}-\alpha a^{2}{\left(k+\frac{\pi }{a}\right)}^{2}}.$$

Shifting the integrals by *π*/*a*, making the integrals dimensionless, and extending the limits to infinity gives

(D8) $$\frac{1}{N}\sum_{k}\frac{1}{E_{1B}-\omega_{k}}=\int_{-\infty }^{\infty }\frac{d\tilde{k}}{2\pi }\frac{1}{E_{1B}-\omega_{0}-\alpha {\tilde{k}}^{2}}\;=-\frac{1}{2\sqrt{\alpha \left(\omega_{0}-E_{1B}\right)}}.$$

Here, we have assumed that *E*_1*B*_ < *ω*_0_. Following the same steps for the remaining integral gives

(D9) $$\frac{1}{N}\sum_{k}\frac{e^{-ika\left(z_{1}-z_{2}\right)}}{E_{1B}-\omega_{k}}=-\frac{\text{cos}\left[\pi \left(z_{1}-z_{2}\right)\right]}{2\sqrt{\alpha \left(\omega_{0}-E_{1B}\right)}}e^{-\sqrt{\left(\omega_{0}-E_{1B}\right)/\alpha }\left|z_{1}-z_{2}\right|}.$$

Here, we use the fact that (*z*_1_ − *z*_2_) is an integer. We then have

(D10) $$E_{1B}a_{1}=\omega_{01;1}a_{1}-\frac{1}{2\sqrt{\alpha \left(\omega_{0}-E_{1B}\right)}}\;\times \left\{a_{1}g_{1}^{2}+a_{2}g_{1}g_{2}\text{ cos}\left[\pi \left(z_{1}-z_{2}\right)\right]e^{-\sqrt{\left(\omega_{0}-E_{1B}\right)/\alpha }\left|z_{1}-z_{2}\right|}\right\}.$$

Repeating these steps for a_2_ gives the following equation for the bound-state energy,

(D11) $$\left(E_{1B}-\omega_{01;1}+\frac{g_{1}^{2}}{2\sqrt{\alpha \left(\omega_{0}-E_{1B}\right)}}\right)\;\times \left(E_{1B}-\omega_{01;2}+\frac{g_{2}^{2}}{2\sqrt{\alpha \left(\omega_{0}-E_{1B}\right)}}\right)\;=\frac{g_{1}^{2}g_{2}^{2}e^{-2\sqrt{\left(\omega_{0}-E_{1B}\right)/\alpha }\left|z_{1}-z_{2}\right|}}{4\alpha \left(\omega_{0}-E_{1B}\right)},$$

where we use the fact that (*z*_1_ − *z*_2_) is an integer again. We note that when the qubits are infinitely far away from each other, we recover the well-known bound-state energy for a single qubit (see, e.g., Ref. [7] or Ref. [13]),

(D12) $$E_{1B}-\omega_{01}=-\frac{g^{2}}{2\sqrt{\alpha \left(\omega_{0}-E_{1B}\right)}}.$$

Generically, Eq. (D11) yields one or two bound states depending on the coupling strength, qubit frequencies, distance between qubits, and *α* [7].

To illustrate this point, we consider the case when *g*_1_ = *g*_2_ = g and *ω*_01;1_ = *ω*_01;2_ = *ω*_01_ [which is relevant to the case in Fig. 3(g) of the main text]. While the coupling strengths are not exactly equal in our experimental system, it is a decent approximation to consider them equal. In this case, we expect a symmetric and an antisymmetric solution, i.e., $a_{1}^{e}=a_{2}^{e}\text{ or }a_{1}^{o}=-a_{2}^{o}$ The difference of the bound-state energy and the energy of the band edge, *E*_1*B*_ − *ω*_0_ = *δE*_1*B*_ < 0, is then given by

(D13) $$\left[\delta E_{1B}-\left(\omega_{01}-\omega_{0}\right)\right]=\Sigma_{\pm }\left(\delta E_{1B}\right)\;=-\frac{g^{2}}{2\sqrt{-\alpha \delta E_{1B}}}\left[1\pm {\left(-1\right)}^{\left|z_{1}-z_{2}\right|}e^{-\sqrt{\left(-\delta E_{1B}\right)/\alpha }\left|z_{1}-z_{2}\right|}\right],$$

where + is for the symmetric state and − is for the antisymmetric state and $\Sigma_{\pm }\left(\delta E_{1B}\right)$ is the self-energy. The condition for the presence of a bound state, as derived in Ref. [7] is $-\left(\omega_{01}-\omega_{0}\right)>\Sigma_{\pm }(0)$.

We explicitly consider the experimentally relevant case when |*z*_1_ − *z*_2_| is odd. In this case, we have $\Sigma_{+}(0)=-\left(g^{2}/2\alpha \right)\left|z_{1}-z_{2}\right|$ and $\Sigma_{-}(0)=-\infty$. For the antisymmetric state, the condition is always satisfied, while for the symmetric state, we only have a bound state if $g>\sqrt{\left[2\alpha \left(\omega_{01}-\omega_{0}\right)\right]/\left|z_{1}-z_{2}\right|}$. We now apply this formalism to our experimental system. For our experimental system, *α* = 1.155 GHz and |*z*_1_ − *z*_2_| = 1. We also take *g* to be the average of the two coupling strengths determined in the previous section, i.e., *g* = (*g*_1_ + *g*_2_)/2 = 0.5275 GHz. Using these numbers, we estimate that we have two bound states for *ω*_01_ − *ω*_0_ < 120 MHz. We remind the reader that this result is only an estimate, as our experimental system is finite and has unequal coupling strengths.

Finally, we investigate the wave function. The ratio of *a*_1_/*a*_2_, which we define to be *η*, is given by

(D14) $$\frac{a_{1}}{a_{2}}=\eta =-\frac{1}{2\sqrt{\alpha \left(\omega_{0}-E_{1B}\right)}}\;\times \frac{g_{1}g_{2}\text{ cos}\left[\pi \left(z_{1}-z_{2}\right)\right]e^{-\sqrt{\left(\omega_{0}-E_{1B}\right)/\alpha }\left|z_{1}-z_{2}\right|}}{E_{1B}-\omega_{01;1}+\frac{g_{1}^{2}}{2\sqrt{\alpha \left(\omega_{0}-E_{1B}\right)}}}.$$

When *g*_1_ = *g*_2_ and *ω*_01;1_ = *ω*_01;2_, we have *a*_1_/*a*_2_ = ±1, i.e., we have symmetric and antisymmetric qubit states. Using the normalization condition, $a_{1}^{2}+a_{2}^{2}=1$, we have $a_{1}^{2}=1/\left[1+\left(1/\eta^{2}\right)\right]$. The photonic part of the wave function is given by

(D15) $$c_{k}=\frac{\text{tan }\phi }{\sqrt{N}}\frac{a_{1}g_{1}e^{ikaz_{1}}+a_{2}g_{2}e^{ikaz_{2}}}{E_{1B}-\omega_{k}}.$$

The condition that $\sum_{k}{\left|c_{k}\right|}^{2}=1$ gives

(D16) $$\frac{1}{\text{tan }\phi^{2}}=\frac{a_{1}^{2}}{N}\sum_{k}\frac{{\left|g_{1}e^{ikaz_{1}}+\frac{1}{\eta }g_{2}e^{ikaz_{2}}\right|}^{2}}{{\left(E_{1B}-\omega_{k}\right)}^{2}}\;=a_{1}^{2}\left(\frac{g_{1}^{2}+\frac{1}{\eta^{2}}g_{2}^{2}}{4{\left(E_{1B}-\omega_{0}\right)}^{3/2}\sqrt{\alpha }}+\frac{g_{1}g_{2}\text{ cos}\left[\pi \left(z_{1}-z_{2}\right)\right]}{2\eta {\left(E_{1B}-\omega_{0}\right)}^{2}\alpha }e^{-\left|z_{1}-z_{2}\right|\sqrt{\left(\omega_{0}-E_{1B}\right)/\alpha }}\left[\left(E_{1B}-\omega_{0}\right)\left|z_{1}-z_{2}\right|+\sqrt{\left(\omega_{0}-E_{1B}\right)\alpha }\right]\right).$$

Fourier transforming *c*_*k*_ gives

(D17) $$c_{j}=\frac{1}{\sqrt{N}}\sum_{k}c_{k}e^{ikaj}=\frac{\text{tan }\phi }{N}\left(a_{1}g_{1}\int_{-\infty }^{\infty }\frac{dk}{2\pi }\frac{e^{ika\left(j-z_{1}\right)}}{E_{1B}-\omega_{k}}+a_{2}g_{2}\int_{-\infty }^{\infty }\frac{dk}{2\pi }\frac{e^{iak\left(j-z_{2}\right)}}{E_{1B}-\omega_{k}}\right)\;=-\text{ tan }\phi \left(a_{1}g_{1}\frac{\text{cos}\left[\pi \left(j-z_{1}\right)\right]}{2\sqrt{\alpha \left(\omega_{0}-E_{1B}\right)}}e^{-\sqrt{\left(\omega_{0}-E_{1B}\right)/\alpha }\left|j-z_{1}\right|}+a_{2}g_{2}\frac{\text{cos}\left[\pi \left(j-z_{2}\right)\right]}{2\sqrt{\alpha \left(\omega_{0}-E_{1B}\right)}}e^{-\sqrt{\left(\omega_{0}-E_{1b}\right)/\alpha }\left|j_{-z_{2}}\right|}\right).$$

We see that the photon is exponentially localized around the qubits with localization length $a\sqrt{\alpha /\left(\omega_{0}-E_{1B}\right)}$ as in the case of a single qubit (see Fig. 7). We remind the reader that we can have two different bound-state energies (one for the symmetric bound state and one for the antisymmetric one), thus two different localization lengths. In other words, the linewidths of the bound states will, in general, be different, with the bound state closer to the band edge having a larger linewidth. In our system, the symmetric bound state, whenever it exists, is closer to the band edge and thus has a larger linewidth.

Finally, we remark that when *g*_1_≈*g*_2_=*g* and *ω*_01;1_≈*ω*_01;2_=*ω*_01_, the symmetric and antisymmetric nature of the bound states can affect transport. Consider the case when the qubits are on the same lattice site. In this limit, the photonic contribution of the antisymmetric wave function will vanish and hence cannot be detected via transport [see Eq. (D17)]. When the qubits are not on the same site, there will be reduced transmission depending on the separation between the qubits and whether or not the state is symmetric or antisymmetric as can be seen explicitly from Eq. (D17). The fact that this effect depends on the separation between qubits can be traced back to the fact that the band edge is at *k* = ±*π* (if the band edge was at *k* = 0, only transport for the antisymmetric state would be reduced). To obtain an estimate for how much transmission will be reduced for the symmetric state, we plot the ratio of linewidths, i.e., ${\left|c_{N}^{+}\right|}^{2}/{\left|c_{N}^{-}\right|}^{2}$ [see Eq. (C9)], for experimentally relevant parameters (|*z*_1_ − *z*_2_| = 1, *N* − *z*_1_ = 7, and *N* − *z*_2_ = 8) in Fig. 8(d). Indeed, we see that the symmetric state has a much smaller linewidth and hence less transmission. This is consistent with the experimental results presented in Fig. 3(e).

### 3. Single-photon bound states: Born-Markov solution

We now briefly compare these exact results to the Born-Markov (BM) approximation. For simplicity, we restrict ourselves to the case when the qubit frequencies and coupling strengths are the same. Using a second-order Schrieffer-Wolff transformation to eliminate the high-energy subspace gives the following effective Hamiltonian for the two qubits,

(D18) $$H_{\text{BM}}=\left(\omega_{01}+\frac{g^{2}}{N}\sum_{k}\frac{1}{\omega_{01}-\omega_{k}}\right)\left({|1\rangle }_{1}{\langle {1|}_{1}+\;|1\rangle }_{2}\langle {1|}_{2}\right)+\frac{g^{2}}{N}\sum_{k}\frac{e^{ika\left(z_{1}-z_{2}\right)}}{\omega_{01}-\omega_{k}}\left({|0\rangle }_{1}{\langle 1\left|{}_{1}\right|1\rangle }_{2}{\langle {0|}_{2}+\;|1\rangle }_{1}{\langle 0\left|{}_{1}\right|0\rangle }_{2}\langle 1|{}_{2}\right)\;=\left(\omega_{01}-\frac{g^{2}}{2\sqrt{\alpha \left(\omega_{0}-\omega_{01}\right)}}\right)\;\left({|1\rangle }_{1}\langle {1|}_{1}+\;{|1\rangle }_{2}\langle 1|{}_{2}\right)\;-\frac{g^{2}}{2\sqrt{\alpha \left(\omega_{0}-\omega_{01}\right)}}\text{cos}\left[\pi \left(z_{1}-z_{2}\right)\right]e^{-\sqrt{\left(\omega_{0}-\omega_{01}\right)/\alpha }\left|z_{1}-z_{2}\right|}\left({|0\rangle }_{1}{\langle 1\left|{}_{1}\right|1\rangle }_{2}{\langle {0|}_{2}+\;|1\rangle }_{1}{\langle 0\left|{}_{1}\right|0\rangle }_{2}\langle 1|{}_{2}\right).$$

We stress that this formula is only valid for *ω*_01_ < *ω*_0_. Diagonalizing *H*_BM_, we have the following eigenvalues,

(D19) $$E_{1}=\omega_{01}-\frac{g^{2}e^{-(1/2)\sqrt{\left(\omega_{0}-\omega_{01}\right)/\alpha }}{}^{\left|z_{1}-z_{2}\right|}}{\sqrt{\alpha \left(\omega_{0}-\omega_{01}\right)}}\;\times \text{sinh}\left(\frac{1}{2}\sqrt{\frac{\omega_{0}-\omega_{01}}{\alpha }}\left|z_{1}-z_{2}\right|\right),$$

(D20) $$E_{2}=\omega_{01}-\frac{g^{2}e^{-(1/2)\sqrt{\left(\omega_{0}-\omega_{01}\right)/\alpha }\left|z_{1}-z_{2}\right|}}{\sqrt{\alpha \left(\omega_{0}-\omega_{01}\right)}}\;\times \text{cosh}\left(\frac{1}{2}\sqrt{\frac{\omega_{0}-\omega_{01}}{\alpha }}\left|z_{1}-z_{2}\right|\right).$$

In the limit where the qubits are infinitely far apart, we recover the standard expression for the dressed qubit frequency,

(D21) $$\omega_{01}^{\prime }=\omega_{01}-\frac{g^{2}}{2\sqrt{\alpha \left(\omega_{0}-\omega_{01}\right)}}.$$

In Fig. 8, we compare the results obtained in the Born-Markov approximation to the exact analytical results. Figure 8(a) shows these results for a single qubit and Fig. 8(b) shows these results for two qubits with |*z*_1_ − *z*_2_| = 1. As expected, the Born-Markov approximation is a good approximation when the qubit frequency is away from the band edge. Closer to the band edge, the Born-Markov approximation fails, particularly for the lower-energy (i.e., antisymmetric) state. Furthermore, by comparing the blue curve in Fig. 8(a) to the experimentally measured bound state in Fig. 1(e), we clearly see that the experiment is not well described by the Born-Markov approximation. We note that the higher energy red line in Fig. 8(b) ends abruptly at *ω*_01_ ≈ 7.920 GHz as there is only one bound state for *ω*_01_ > *ω*_0_ + 0.120 GHz.

We now analytically show that the Born-Markov approximation is excellent for one of the dressed states close to the band edge. We begin by expanding Eq. (D13) (for the symmetric case, when |*z*_1_ − *z*_2_| = 1) in the limit that *ω*_0_ − *E*_1*B*_ ≪ *α*, i.e., when the bound-state energy is close the band edge. This gives

(D22) $$E_{1B}=\omega_{01}-\frac{g^{2}}{2\alpha }\left[1+O\left(\frac{E_{1B}-\omega_{0}}{\alpha }\right)\right].$$

Now comparing to the Born-Markov solution for *E*_1_ in Eq. (D19) around *ω*_0_ ≈ *ω*_01_, we have *E*_1_ ≈ *ω*_01_ − (*g*^2^=2*α*). We thus see that one of the dressed states (the symmetric one) is well captured by the Born-Markov approximation, while the other is not as seen in Fig. 8(b).

### 4. Single-photon transport via the bound state

As discussed, a bound state mediates transport across the crystal, at the otherwise forbidden frequencies inside the band gap, via the overlap of the photonic mode with the ends of the crystal. However, unlike a cavity mode which accommodates many photons (of the same frequency) due to its harmonic nature, the bound state inherits an anharmonic level structure from the qubit. This will result in single-photon, blockaded transport. For a definitive confirmation, we measure the second-order autocorrelation of the transmitted component of a weak, resonant, continuous drive.

In Fig. 2(c), we plot the emission spectrum of the resonantly driven bound state for low drive amplitudes. Here, we see the familiar Mollow triplet structure featuring sidebands that are linearly displaced from the center peak with increasing drive amplitude. We measure second-order autocorrelation [Fig. 2(c) inset] for a drive amplitude below the threshold for incoherent triplet emission such that the qubit is not saturated by the drive. This measurement (see [36,51–54] for concept) was made possible by a TWPA (MIT Lincoln Labs) to improve SNR and a GPU (CUDA-Matlab) for significant computational speed-up.

## APPENDIX E: EMISSION THEORY

In this Appendix, we discuss the theoretical modeling of the emission spectrum of a resonantly driven bound state. Unfortunately, due to the large dimension of the Hilbert space, we found that a direct approach of calculating the emission spectrum using the master equation for our sixteen-unit-cell system is not numerically feasible. Instead, we diagonalize the Hamiltonian and investigate energy differences. While this approach does not predict the widths and the driving-strength-dependent intensities of the sidebands, it does predict the frequencies of the sidebands. The Hamiltonian of a single transmon qubit with a driving frequency *ω*_d_ equal to the bound-state frequency is given by (in the rotating frame) [55]

(E1) $$H=\sum_{i,j}\left(J_{i,j}-\omega_{d}\delta_{i,j}\right)a_{i}^{†}a_{j}+g_{2}\;\left[a_{z_{2}}^{†}(|0\rangle \;\langle 1|+\sqrt{2}|1\rangle \;\times \langle 2|+\;\sqrt{3}|2\rangle \;\langle 3|+\;\sqrt{4}|3\rangle \;\langle 4|)+\text{H.c.}\right]\;+\sum_{n=0}^{4}\left(\omega_{0n}-n\omega_{d}\right)|n\rangle \;\langle n|+\;\Omega [(|0\rangle \;\langle 1|+\;\sqrt{2}|1\rangle \;\times \langle 2|+\sqrt{3}|2\rangle \;\langle 3|+\sqrt{4}|3\rangle \;\langle 4|)+\text{H}.\text{c}.],$$

where Ω is the bare Rabi frequency of the drive and is the only unknown parameter. We stress that *ω*_d_ is not at the bare qubit frequency but at the frequency of the bound state. In the presence of a drive, the excitation number is no longer conserved, and thus, to make progress, one must implement a cut-off. In our numerical simulations, we have implemented a cut-off of five transmon qubit levels and three photons. Diagonalizing the system, we take the differences of the eigenvalues of states corresponding to large occupation of atomic states with no photons.

We now compare the results obtained by diagonalizing Eq. (E1) to the results obtained by driving a dressed-transmon qubit (without explicitly including the photonic crystal). The latter approach was used in Ref. [13]. The Hamiltonian for the dressed-transmon qubit is

(E2) $$H=\sum_{n=0}^{4}\left({\tilde{\omega }}_{0n}-n\omega_{d}\right)|\tilde{n}\rangle \;\langle \tilde{n}|+\tilde{\Omega }(|\tilde{0}\rangle \langle \tilde{1}|+\sqrt{2}|\tilde{1}\rangle \;\times \langle \tilde{2}|+\sqrt{3}|\tilde{2}\rangle \;\langle \tilde{3}|+\sqrt{4}|\tilde{3}\rangle \;\langle \tilde{4}|+\text{H.c.}).$$

Here, ${\tilde{\omega }}_{0n}$ are the dressed-transmon qubit frequencies, $\omega_{d}={\tilde{\omega }}_{01}$, and $\tilde{\Omega }$ is the Rabi frequency seen by the dressed-transmon qubit. If the exact wave function for the bound state is $|\psi \rangle =\text{cos }\theta |1\rangle |0\rangle +\text{sin }\theta \sum_{k}c_{k}a_{k}^{†}|0\rangle |0\rangle$ with $\sum_{k}{\left|c_{k}\right|}^{2}=1$ (here, our basis states are labeled as |qubit〉|photon〉), we have $\tilde{\Omega }\approx \Omega \text{ cos }\theta$, where *θ* is given by

(E3) $${\text{tan}}^{2}\theta =\frac{g^{2}}{N}\sum_{k}\frac{1}{{\left(E_{1B}-\omega_{k}\right)}^{2}},$$

which, for an infinite system, simplifies to

(E4) $${\text{tan}}^{2}\theta =\frac{g^{2}}{4}\frac{1}{{\left(E_{1B}-\omega_{0}\right)}^{3/2}\sqrt{\alpha }}.$$

When the bound state is approximately at 7.59 GHz, we find cos *θ* ≈ 0.68 for our finite system. Figure 9 compares the results obtained from Eqs. (E1) and (E2). We see that, upon taking into account the reduction of the matrix element due to the dressing, the two methods agree. We also see [Fig. 9(b)] that, as expected, as the bare qubit frequency moves deeper into the gap, *θ* approaches zero and $\tilde{\Omega }$ approaches Ω. In Fig. 9, we have used the anharmonicity value predicted by theory. In Fig. 2(c) of the main text, we use the experimental values of anharmonicity, which is given by ${\tilde{\omega }}_{02}-2\omega_{d}=-0.11$ GHz.

We assume that the feature around 7.22 GHz in Fig. 2(c) of the main text is approximately ${\tilde{\omega }}_{23}$ (which ${\tilde{\omega }}_{03}-3\omega_{d}=-0.48$ GHz). This assumption is in decent agreement (around 50 MHz off) with results obtained by diagonalizing the full system when the bound state is at 7.59 GHz. This 50-MHz disagreement can be traced back to the 20-MHz disagreement in $\tilde{\Delta }$, the dressed anharmonicity, between theory and experiment when the bound state is at 7.59 GHz (if $\tilde{\Delta }$ is off by 20 MHz, level |3〉 is expected to be off from its value by around 3 times this amount as ${\tilde{\omega }}_{03}\approx 3{\tilde{\omega }}_{01}-3\tilde{\Delta }$). Finally, we find that ${\tilde{\omega }}_{04}-4\omega_{d}=-1.78$ GHz [this value is also consistent with results obtained by diagonalizing Eq. (E1)] matches the experimental data well as seen by overlaying the theoretical data from the dressed qubit with the experiential data [Fig. 2(c) of main text]. In particular, we have captured the feature that appears around 7.22 GHz and −10 dB. The unique bending of this feature can be traced back to the fact that the level structure of the dressed qubit [Eq. (E2)] does not behave like a normal transmon due to the strong coupling to the photonic crystal.

## APPENDIX F: MULTIPHOTON THEORY

### 1. Two-photon bound state

In this section, we discuss the two-photon bound state. For a single transmon qubit, the most general two-excitation wave function is

(F1) $$|\psi_{2B}\rangle =b|2\rangle |0\rangle +\sum_{i}d_{i}a_{i}^{†}|1\rangle |0\rangle +\sum_{i>j}f_{i,j}a_{i}^{†}a_{j}^{†}|0\rangle |0\rangle \;+\sum_{i}f_{i,i}\frac{{\left(a_{i}^{†}\right)}^{2}}{\sqrt{2}}|0\rangle |0\rangle .$$

Here, the basis states are labeled as |transmon〉|photon〉. For *i* < *j*, it is convenient to define *f*_*i*,*j*_ = *f*_*i*,*j*_. In Fig. 10 we plot |*d*_*i*_|^2^ and |*f*_*i*,*j*_|^2^, which are obtained via exact diagonalization of the two-excitation sector for 16 sites [56]. We observe that the photons are localized around the qubit. In Fig. 10(c), we plot the populations of transmon qubit levels in the two-excitation ground state of *H*_tot_, which are given by

(F2) $${\left|\langle 0|\psi_{2B}\rangle \right|}^{2}=|b{|}^{2},\;{\left|\langle 1|\psi_{2B}\rangle \right|}^{2}=\sum_{i}{\left|d_{i}\right|}^{2},\;{\left|\langle 2|\psi_{2B}\rangle \right|}^{2}=\sum_{i\geq j}{\left|f_{i,j}\right|}^{2},$$

to illustrate which terms in Eq. (F1) are important for a given bare qubit frequency. For example, when the bare qubit frequency is deep in the gap, the ground state of the two-excitation sector is mostly in the |2〉|0〉 state as seen in Fig. 10(c). Upon increasing the bare qubit frequency (while still in the band gap), the population of |1〉 increases, while the population of |0〉 stays relatively small. This is because two photons must be exchanged to couple the dominate |2〉|0〉 state and any two-photon state and there are no terms in *H*_tot_ that directly exchange two photons, thus making it a higher-order process. On the other hand, coupling |2〉|0〉 and $a_{i}^{†}|1\rangle |0\rangle$ only requires exchanging one photon. When the bare qubit frequency is at or near the band edge, each transmon qubit level in Eq. (F1) contributes significantly to the bound-state wave function.

Unfortunately, we are unaware of an exact solution similar to the one in Sec. D 2. To make analytical progress, we assume *f*_*i*,*j*_ = 0 which is a valid approximation when the bare qubit frequency is deep in the band gap [as seen in Fig. 10(c)]. Solving the eigenvalue equation, $H|{\tilde{\psi }}_{2B}\rangle =E_{2B}|{\tilde{\psi }}_{2B}\rangle$, for the following wave function ansatz (in momentum space),

(F3) $$|{\tilde{\psi }}_{2B}\rangle =\tilde{b}|2\rangle |0\rangle +\sum_{k}{\tilde{d}}_{k}a_{k}^{†}|1\rangle |0\rangle ,$$

yields the following equations:

(F4) $$\tilde{b}E_{2B}=\omega_{02}\tilde{b}+\frac{\sqrt{2}g}{\sqrt{n}}\sum_{k}e^{ikaz_{2}}{\tilde{d}}_{k},$$

(F5) $${\tilde{d}}_{k}E_{2B}=\left(\omega_{k}+\omega_{01}\right){\tilde{d}}_{k}+\frac{\sqrt{2}g}{\sqrt{N}}e^{-ikaz_{2}}\tilde{b}.$$

These are similar to equations for the single-photon case. Thus, we have

(F6) $$E_{2B}-\omega_{02}=-\frac{g^{2}}{\sqrt{\omega_{0}+\omega_{01}-E_{2B}}}$$

and

(F7) $$d_{j}\propto \frac{\sqrt{2}\tilde{b}}{N}\sum_{k}\frac{e^{ika\left(j-z_{2}\right)}}{E_{2B}-\omega_{01}-\omega_{k}}\;=-\frac{\sqrt{2}\tilde{b}\text{ cos}\left[\pi \left(j-z_{2}\right)\right]}{2\sqrt{\alpha \left(\omega_{0}+\omega_{01}-E_{2B}\right)}}e^{-\sqrt{\left(\omega_{0}+\omega_{01}-E_{2B}\right)/\alpha }\left|j-z_{2}\right|}.$$

We see that the photon is localized around the transmon qubit, consistent with the exact finite-size numerical results seen in Fig. 10(a). We note this ansatz breaks down when the bare qubit frequency is near the passband as the states become more photonic, in which case we can no longer neglect *f*_*i*,*j*_.

### 2. Two-photon avoided crossing

In this section, we discuss the two-photon avoided crossing seen in Fig. 4 of the main text. We assume the two transmon qubits have equal coupling strengths and equal bare frequencies. To begin, we first write the total Hamiltonian in the two-excitation sector (in the rotating frame), using a different notation,

(F8) $$H_{2}=H_{0x}+H_{0y}+H_{1x}+H_{1y},$$

where

(F9) $$H_{0x}=\sum_{k}\left(\omega_{k}+\omega_{12}\right)(|0,1;k\rangle \;\langle 0,1;k|+|1,0;k\rangle \;\langle 1,0;k|),$$

(F10) $$H_{0y}=\sum_{k,p}\left(\omega_{k}+\omega_{p}-\omega_{02}\right)|0,0;k,p\rangle \;\langle 0,0;k,p|,$$

(F11) $$H_{1x}=\frac{\sqrt{2}g}{\sqrt{N}}\sum_{k}\left(e^{ikaz_{1}}|1,0;k\rangle \langle 2,0;0\left|\;+e^{ikaz_{2}}\right|0,1;k\rangle \langle 0,2;0|+\text{H.c.}\right),$$

(F12) $$H_{1y}=\frac{g}{\sqrt{N}}\sum_{k,p;k\neq p}\left(e^{ipaz_{1}}|0,0;k,p\rangle \langle 1,0;k\left|\;+\;e^{ipaz_{2}}\right|0,0;k,p\rangle \langle 0,1;k|+\text{H.c.}\right)\;+\frac{\sqrt{2}g}{\sqrt{N}}\sum_{k}\left(e^{ikaz_{1}}|0,0;k,k\rangle \langle 1,0;k\left|\;+e^{ikaz_{2}}\right|0,0;k,k\rangle \langle 0,1;k|+\text{H.c.}\right).$$

Here, the basis states are labeled as |transmo_1_; transmo_2_; photonic field〉. To proceed, we neglect the second line in *H*_1*y*_ as there are many photonic modes, thus the probability of both photons going into the same mode is negligible. Using a unitary Schrieffer-Wolff transformation, we find the fourth-order term in coupling strength *g* to be

(F13) $$H_{4}=\frac{1}{2}H_{1x}{\tilde{H}}_{0}\left({\tilde{H}}_{0}H_{1x}^{2}+H_{1x}^{2}{\tilde{H}}_{0}\right){\tilde{H}}_{0}H_{1x}-H_{1x}{\tilde{H}}_{0}H_{1y}{\tilde{H}}_{0}H_{1y}{\tilde{H}}_{0}H_{1x},$$

where *H*_0_ = *H*_0*x*_ + *H*_0*y*_ and ${\tilde{H}}_{0}=H_{0}^{-1}$ (here, $H_{0}^{-1}$ is taken to be zero outside the support of *H*_0_). We are only interested in terms that involve interactions between the |0, 2; 0〉 and |2, 0; 0〉 states, i.e., terms like |2, 0; 0〉〈0, 2; 0|. Only the last term in Eq. (F13) contributes such a term. Ignoring contributions of the last term that are diagonal in the {|0, 2; 0〉, |2, 0; 0〉} basis, the effective interaction between the |2, 0〉 and the |0; 2〉 states is (after projecting out the photonic degrees of freedom),

(F14) $$H_{|2,0\rangle ↔|0,2\rangle }=-\frac{4g^{4}}{N^{2}}|2,0\rangle \;\langle 0,2|\sum_{k,p}\frac{e^{i(k+p)a\left(z_{2}-z_{1}\right)}}{\left(\omega_{k}+\omega_{p}-\omega_{02}\right)\left(\omega_{k}-\omega_{12}\right)}\left(\frac{1}{\omega_{k}-\omega_{12}}+\frac{1}{\omega_{p}-\omega_{12}}\right)+\text{H.c.}\;=-4g^{4}|2,0\rangle \;\langle 0,2|\int_{-\infty }^{\infty }\frac{d\tilde{p}}{2\pi }\int_{-\infty }^{\infty }\frac{d\tilde{k}}{2\pi }\frac{e^{i(\tilde{k}+\tilde{p})\left(z_{2}-z_{1}\right)}}{\left[\alpha \left({\tilde{k}}^{2}+{\tilde{p}}^{2}\right)-2\omega_{0}-\omega_{02}\right]\left(\alpha {\tilde{k}}^{2}+\omega_{0}-\omega_{12}\right)}\;\times \left(\frac{1}{\alpha {\tilde{k}}^{2}+\omega_{0}-\omega_{12}}+\frac{1}{\alpha {\tilde{p}}^{2}+\omega_{0}-\omega_{12}}\right)+\text{H.c.}$$

The dimensionless $\tilde{p}$ integral can be evaluated exactly. Doing so, we have

(F15) $$H_{|2,0\rangle ↔|0,2\rangle }=-2g^{4}|2,0\rangle \;\langle 0,2|\int_{-\infty }^{\infty }\frac{d\tilde{k}}{2\pi }e^{i\tilde{k}\left(z_{2}-z_{1}\right)}\left[\frac{e^{\left(-\left|z_{2}-z_{1}\right|/\sqrt{\alpha }\right)\sqrt{2\omega_{0}+\alpha {\tilde{k}}^{2}-\omega_{02}}}}{\sqrt{\alpha }\sqrt{2\omega_{0}+\alpha {\tilde{k}}^{2}-\omega_{02}}}\frac{1}{{\left(\alpha {\tilde{k}}^{2}+\omega_{0}-\omega_{12}\right)}^{2}}-\frac{1}{\left(\alpha {\tilde{k}}^{2}+\omega_{0}-\omega_{12}\right)}\frac{1}{\omega_{0}+\omega_{12}-\omega_{02}+\alpha {\tilde{k}}^{2}}\left(\frac{e^{\left(\left|z_{2}-z_{1}\right|/\sqrt{\alpha }\right)\sqrt{2\omega_{0}+\alpha {\tilde{k}}^{2}-\omega_{02}}}}{\sqrt{\alpha }\sqrt{2\omega_{0}+\alpha {\tilde{k}}^{2}-\omega_{02}}}-\frac{e^{\left(-\left|z_{2}-z_{1}\right|/\sqrt{\alpha }\right)\sqrt{\omega_{0}-\omega_{12}}}}{\sqrt{\alpha }\sqrt{\omega_{0}-\omega_{12}}}\right)\right]+\text{H.c.}$$

Here, we have assumed that 2*ω*_0_ > *ω*_02_ and *ω*_0_ > *ω*_12_. These conditions are satisfied in the regime where we experimentally observe the two-photon avoided crossing. We are interested in determining how the interaction decays as a function of distance. Unfortunately, we are unaware of how to analytically evaluate the first two terms (the third term can be evaluated exactly). However, the integrand decreases exponentially as a function of k for the first two terms. Thus, it is reasonable to take the integrand to be a constant value (i.e., the integrand value at *k* = 0) over a small window, *δk*, about $\tilde{k}=0$ and zero otherwise. This small window is taken to be the momentum value for which the argument in the exponential equals one, i.e., $\delta k=2\sqrt{\left(1/{\left|z_{2}-z_{1}\right|}^{2}\right)-\left[\left(2\omega_{0}-\omega_{02}\right)/\alpha \right]}$. Evaluating the remaining integral gives

(F16) $$H_{|2,0\rangle ↔|0,2\rangle }\approx -2g^{4}\frac{\left|2,0\rangle \;\langle 0,2\right|}{\left(\omega_{0}-\omega_{12}\right)}\frac{\delta k}{2\pi }\frac{e^{-\left|z_{2}-z_{1}\right|\sqrt{\left(2\omega_{0}-\omega_{02}\right)/\alpha }}}{\sqrt{\alpha }\sqrt{2\omega_{0}-\omega_{02}}}\left(\frac{1}{\left(\omega_{0}-\omega_{12}\right)}-\frac{1}{\omega_{0}+\omega_{12}-\omega_{02}}\right)\;+g^{4}|2,0\rangle \;\langle 0,2|\frac{1}{\sqrt{\alpha }\left(\omega_{02}-2\omega_{12}\right)}\left(\frac{e^{-\left|z_{2}-z_{1}\right|\sqrt{\left(\omega_{0}-\omega_{12}\right)/\alpha }}}{\sqrt{\omega_{0}-\omega_{12}}}-\frac{e^{-\left|z_{2}-z_{1}\right|\sqrt{\left(\omega_{0}+\omega_{12}-\omega_{02}\right)/\alpha }}}{\sqrt{\omega_{0}+\omega_{12}-\omega_{02}}}\right)\frac{e^{-\left(\left|z_{2}-z_{1}\right|/\sqrt{\alpha }\right)\sqrt{\omega_{0}-\omega_{12}}}}{\sqrt{\alpha }\sqrt{\omega_{0}-\omega_{12}}}+\text{H.c.}$$

We see that every term decays exponentially as a function of |*z*_2_ − *z*_1_|, thus the effective interaction between the |2, 0〉 and the |0, 2〉 states decays exponentially as well.

## References

[1] Wang J and John S, Quantum Optics of Localized Light in a Photonic Band Gap, Phys. Rev. B 43, 12772(1991). 10.1103/physrevb.43.12772 9997091

[2] John S, Quantum Electrodynamics of Localized Light, Physica (Amsterdam) 175B, 87(1991).

[3] Kofman AG, Kurizki G, and Sherman B, Spontaneous and Induced Atomic Decay in Photonic Band Structures, J. Mod. Opt 41, 353(1994).

[4] John S and Quang T, Spontaneous Emission near the Edge of a Photonic Band Gap, Phys. Rev. A 50, 1764(1994). 9911069 10.1103/physreva.50.1764

[5] John S and Quang T, Photon-Hopping Conduction and Collectively Induced Transparency in a Photonic Band Gap, Phys. Rev. A 52, 4083(1995). 9912723 10.1103/physreva.52.4083

[6] Shahmoon E and Kurizki G, Nonradiative Interaction and Entanglement between Distant Atoms, Phys. Rev. A 87, 033831(2013).

[7] Calajó G, Ciccarello F, Chang D, and Rabl P, Atom-Field Dressed States in Slow-Light Waveguide QED, Phys. Rev. A 93, 033833(2016).

[8] Joannopoulos J, Johnson S, Winn JN, and Meade R, Photonic Crystals: Molding the Flow of Light (Princeton University Press, Princeton, New Jersey, 2008).

[9] Goban A, Hung C-L, Yu S-P, Hood J, Muniz J, Lee J, Martin M, McClung A, Choi K, Chang D, Painter O, and Kimble H, Atom–Light Interactions in Photonic Crystals, Nat. Commun 5, 3808(2014). 24806520 10.1038/ncomms4808

[10] Goban A, Hung CL, Hood JD, Yu SP, Muniz JA, Painter O, and Kimble HJ, Superradiance for Atoms Trapped along a Photonic Crystal Waveguide, Phys. Rev. Lett 115, 063601(2015). 26296116 10.1103/PhysRevLett.115.063601

[11] González-Tudela A, Hung C-L, Chang DE, Cirac JI, and Kimble HJ, Quantum Physics Subwavelength Vacuum Lattices and Atom-Atom Interactions in Photonic Crystals, Nat. Photonics 9, 320(2015).

[12] Hood JD, Goban A, Asenjo-Garcia A, Lu M, Yu SP, Chang DE, and Kimble HJ, Atom–Atom Interactions Around the Band Edge of a Photonic Crystal Waveguide, Proc. Natl. Acad. Sci. U.S.A 113, 10507(2016). 27582467 10.1073/pnas.1603788113 PMC5035845

[13] Liu Y and Houck AA, Quantum Electrodynamics near a Photonic Bandgap, Nat. Phys 13, 48(2017).

[14] Munro E, Kwek LC, and Chang DE, Optical Properties of an Atomic Ensemble Coupled to a Band Edge of a Photonic Crystal Waveguide, New J. Phys 19, 083018(2017).

[15] Shi T, Wu YH, Gonzalez-Tudela A, and Cirac JI, Bound States in Boson Impurity Models, Phys. Rev. X 6, 021027(2016).

[16] Hung CL, González-Tudela A, Cirac JI, and Kimble HJ, Quantum Spin Dynamics with Pairwise-Tunable, Long-Range Interactions, Proc. Natl. Acad. Sci. U.S.A 113, E4946(2016). 27496329 10.1073/pnas.1603777113 PMC5003233

[17] Douglas JS, Habibian H, Hung C-L, Gorshkov AV, Kimble HJ, and Chang DE, Quantum Many-Body Models with Cold Atoms Coupled to Photonic Crystals, Nat. Photonics 9, 326(2015).

[18] Sánchez-Burillo E, Zueco D, Martín-Moreno L, and García-Ripoll JJ, Dynamical Signatures of Bound States in Waveguide QED, Phys. Rev. A 96, 023831(2017).

[19] Asenjo-Garcia A, Hood JD, Chang DE, and Kimble HJ, Atom-Light Interactions in Quasi-One-Dimensional Nanostructures: A Green’s-Function Perspective, Phys. Rev. A 95, 033818(2017).

[20] Kollár AJ, Papageorge AT, Vaidya VD, Guo Y, Keeling J, and Lev BL, Supermode-Density-Wave-Polariton Condensation with a Bose–Einstein Condensate in a Multimode Cavity, Nat. Commun 8, 14386(2017). 28211455 10.1038/ncomms14386 PMC5321730

[21] Vaidya VD, Guo Y, Kroeze RM, Ballantine KE, Kollár AJ, Keeling J, and Lev BL, Tunable-Range, Photon-Mediated Atomic Interactions in Multimode Cavity QED, Phys. Rev. X 8, 011002(2018).

[22] Zhou L, Gong ZR, Liu Y, Sun CP, and Nori F, Controllable Scattering of a Single Photon inside a One-Dimensional Resonator Waveguide, Phys. Rev. Lett 101, 100501(2008). 18851197 10.1103/PhysRevLett.101.100501

[23] Zhou L, Dong H, Liu Y, Sun CP, and Nori F, Quantum Supercavity with Atomic Mirrors, Phys. Rev. A 78, 063827(2008).

[24] Liao J, Gong ZR, Zhou L, Liu Y, Sun CP, and Nori F, Controlling the Transport of Single Photons by Tuning the Frequency of Either One or Two Cavities in an Array of Coupled Cavities, Phys. Rev. A 81, 042304(2010).

[25] Majer J, Chow JM, Gambetta JM, Koch J, Johnson BR, Schreier JA, Frunzio L, Schuster DI, Houck AA, Wallraff A, Blais A, Devoret MH, Girvin SM, and Schoelkopf RJ, Coupling Superconducting Qubits via a Cavity Bus, Nature (London) 449, 443(2007). 17898763 10.1038/nature06184

[26] DiCarlo L, Chow JM, Gambetta JM, Bishop LS, Johnson BR, Schuster DI, Majer J, Blais A, Frunzio L, Girvin SM, and Schoelkopf RJ, Demonstration of Two-Qubit Algorithms with a Superconducting Quantum Processor, Nature (London) 460, 240(2009). 19561592 10.1038/nature08121

[27] van Loo AF, Fedorov A, Lalumiere K, Sanders BC, Blais A, and Wallraff A, Photon-Mediated Interactions Between Distant Artificial Atoms, Science 342, 1494(2013). 24231805 10.1126/science.1244324

[28] Nigg SE, Paik H, Vlastakis B, Kirchmair G, Shankar S, Frunzio L, Devoret MH, Schoelkopf RJ, and Girvin SM, Black-Box Superconducting Circuit Quantization, Phys. Rev. Lett 108, 240502(2012). 23004246 10.1103/PhysRevLett.108.240502

[29] Biondi M, Schmidt S, Blatter G, and Türeci HE, Self-Protected Polariton States in Photonic Quantum Metamaterials, Phys. Rev. A 89, 025801(2014).

[30] Pozar D, Microwave Engineering Fourth Edition (Wiley, Hoboken, New Jersey, 2012), pp. 1–756.

[31] Shen JT and Fan S, Coherent Single Photon Transport in a One-Dimensional Waveguide Coupled with Superconducting Quantum Bits, Phys. Rev. Lett 95, 213001(2005). 16384136 10.1103/PhysRevLett.95.213001

[32] Shen JT and Fan S, Theory of Single-Photon Transport in a Single-Mode Waveguide. I. Coupling to a Cavity Containing a Two-Level Atom, Phys. Rev. A 79, 023837(2009).

[33] Hafezi M, Chang DE, Gritsev V, Demler E, and Lukin MD, Quantum Transport of Strongly Interacting Photons in a One-Dimensional Nonlinear Waveguide, Phys. Rev. A 85, 013822(2012).

[34] Astafiev O, Zagoskin AM, Abdumalikov AA, Pashkin YA, Yamamoto T, Inomata K, Nakamura Y, and Tsai JS, Resonance Fluorescence of a Single Artificial Atom, Science 327, 840(2010). 20150495 10.1126/science.1181918

[35] Abdumalikov AA, Astafiev OV, Pashkin YA, Nakamura Y, and Tsai JS, Dynamics of Coherent and Incoherent Emission from an Artificial Atom in a 1D Space, Phys. Rev. Lett 107, 043604(2011). 21867005 10.1103/PhysRevLett.107.043604

[36] Hoi IC, Palomaki T, Lindkvist J, Johansson G, Delsing P, and Wilson CM, Generation of Nonclassical Microwave States Using an Artificial Atom in 1D Open Space, Phys. Rev. Lett 108, 263601(2012). 23004976 10.1103/PhysRevLett.108.263601

[37] Koshino K, Terai H, Inomata K, Yamamoto T, Qiu W, Wang Z, and Nakamura Y, Observation of the Three-State Dressed States in Circuit Quantum Electrodynamics, Phys. Rev. Lett 110, 263601(2013). 23848874 10.1103/PhysRevLett.110.263601

[38] Koch J, Yu T, Gambetta J, Houck A, Schuster D, Majer J, Blais A, Devoret M, Girvin S, and Schoelkopf R, Charge-Insensitive Qubit Design Derived from the Cooper Pair Box, Phys. Rev. A 76, 042319(2007).

[39] Bellomo B, Franco RL, Maniscalco S, and Compagno G, Entanglement Trapping in Structured Environments, Phys. Rev. A 78, 060302(2008).

[40] Tong QJ, An JH, Luo HG, and Oh CH, Mechanism of Entanglement Preservation, Phys. Rev. A 81, 052330(2010).

[41] Yang WL, An JH, Zhang C, Feng M, and Oh CH, Preservation of Quantum Correlation between Separated Nitrogen-Vacancy Centers Embedded in Photonic-Crystal Cavities, Phys. Rev. A 87, 022312(2013).

[42] Beaudoin F, Silva MP, Dutton Z, and Blais A, First-Order Sidebands in Circuit QED Using Qubit Frequency Modulation, Phys. Rev. A 86, 022305(2012).

[43] Strand JD, Ware M, Ohki TA, Johnson BR, Blais A, and Plourde BLT, First-Order Sideband Transitions with Flux-Driven Asymmetric Transmon Qubits, Phys. Rev. B 87 220505(R) (2013).

[44] Mckay DC, Filipp S, Mezzacapo A, Magesan E, Chow JM, and Gambetta JM, Universal Gate for Fixed-Frequency Qubits via a Tunable Bus, Phys. Rev. Applied 6, 064007(2016).

[45] Naik RK, Leung N, Chakram S, Groszkowski P, Lu Y, Earnest N, McKay DC, Koch J, and Schuster DI, Random Access Quantum Information Processors Using Multimode Circuit Quantum Electrodynamics, Nat. Commun 8, 1904(2017). 29199271 10.1038/s41467-017-02046-6 PMC5712528

[46] Raftery J, Vrajitoarea A, Zhang G, Leng Z, Srinivasan SJ, and Houck AA, Direct Digital Synthesis of Microwave Waveforms for Quantum Computing, arXiv:1703.00942v1.

[47] Lund-Hansen T, Stobbe S, Julsgaard B, Thyrrestrup H, Sunner T, Kamp M, Forchel A, and Lodahl P, Experimental Realization of Highly Efficient Broadband Coupling of Single Quantum Dots to a Photonic Crystal Waveguide, Phys. Rev. Lett 101, 113903(2008). 18851282 10.1103/PhysRevLett.101.113903

[48] Yu SP, Hood JD, Muniz JA, Martin MJ, Norte R, Hung CL, Meenehan SM, Cohen JD, Painter O, and Kimble HJ, Nanowire Photonic Crystal Waveguides for Single-Atom Trapping and Strong Light-Matter Interactions, Appl. Phys. Lett 104, 111103(2014).

[49] Bronn NT, Liu Y, Hertzberg JB, Córcoles AD, Houck AA, Gambetta JM, and Chow JM, Broadband Filters for Abatement of Spontaneous Emission in Circuit Quantum Electrodynamics, Appl. Phys. Lett 107, 172601(2015).

[50] The detuned qubit will shift the frequency of the bound state. To account for this effect, we estimate the ratio of coupling strengths. When the bare qubit frequency is resonant with the band edge, the ratio of coupling strengths can be estimated as *g*_1_/*g*_2_ ≈ (Δ_1_/Δ_2_)^3/4^ [see Ref. [13] or Eq. (D12)], where Δ_*i*_ is the frequency difference between the band edge and the bound state for the *i*th qubit. Experimental data indicate Δ_2_ ≈ 250 MHz and Δ_2_ − Δ_1_ ≈ 25 MHz, which corresponds to *g*_1_/*g*_2_ = 0.93. Upon fitting the data, we estimate that *g*_2_ = 0.55 GHz (and hence, *g*_1_ ≈ 0.512 GHz). Given that our value of *g*_1_ is only an estimate, one may wonder about the effect of fine-tuning the coupling strength of the far detuned qubit. We find that even if one changes the coupling strength of the detuned qubit, *g*_1_, by 25 MHz away from 0.512 GHz, the bound statewill only move around half a MHz. In other words, the effect of any fine-tuning of *g*_1_ can be safely ignored.

[51] Da Silva MP, Bozyigit D, Wallraff A, and Blais A, Schemes for the Observation of Photon Correlation Functions in Circuit QED with Linear Detectors, Phys. Rev. A 82, 043804(2010).

[52] Eichler C, Bozyigit D, Lang C, Steffen L, Fink J, and Wallraff A, Experimental Tomographic State Reconstruction of Itinerant Microwave Photons, Phys. Rev. Lett 106, 220503(2011). 21702587 10.1103/PhysRevLett.106.220503

[53] Lang C, Bozyigit D, Eichler C, Steffen L, Fink JM, Abdumalikov AA, Baur M, Filipp S, da Silva MP, Blais A, and Wallraff A, Observation of Resonant Photon Blockade at Microwave Frequencies Using Correlation Function Measurements, Phys. Rev. Lett 106, 243601(2011). 21770569 10.1103/PhysRevLett.106.243601

[54] Bozyigit D, Lang C, Steffen L, Fink JM, Eichler C, Baur M, Bianchetti R, Leek PJ, Filipp S, da Silva MP, Blais A, and Wallraff A, Antibunching of Microwave-Frequency Photons Observed in Correlation Measurements Using Linear Detectors, Nat. Phys 7, 154(2011).

[55] In numerical simulations with a drive, we only consider one transmon qubit. This results in differences of up to 10 MHz when compared to simply detuning one of the transmon qubits.

[56] As a technical aside, we note that our two-excitation numerical simulations involve two transmon qubits unless explicitly noted. For this section, the transmon qubit on site eight is detuned to 4.25 GHz for our numerical simulations.

